# Computational Evaluation of Nucleotide Insertion Opposite Expanded and Widened DNA by the Translesion Synthesis Polymerase Dpo4

**DOI:** 10.3390/molecules21070822

**Published:** 2016-06-23

**Authors:** Laura Albrecht, Katie A. Wilson, Stacey D. Wetmore

**Affiliations:** Department of Chemistry and Biochemistry, University of Lethbridge, 4401 University Drive West, Lethbridge Alberta, AB T1K 3M4, Canada; laura.albrecht@uleth.ca (L.A.); ka.wilson@uleth.ca (K.A.W.)

**Keywords:** expanded DNA, xDNA, widened DNA, yDNA, DNA replication, translesion synthesis, bypass polymerase, Dpo4, molecular dynamics

## Abstract

Expanded (x) and widened (y) deoxyribose nucleic acids (DNA) have an extra benzene ring incorporated either horizontally (xDNA) or vertically (yDNA) between a natural pyrimidine base and the deoxyribose, or between the 5- and 6-membered rings of a natural purine. Far-reaching applications for (x,y)DNA include nucleic acid probes and extending the natural genetic code. Since modified nucleobases must encode information that can be passed to the next generation in order to be a useful extension of the genetic code, the ability of translesion (bypass) polymerases to replicate modified bases is an active area of research. The common model bypass polymerase DNA polymerase IV (Dpo4) has been previously shown to successfully replicate and extend past a single modified nucleobase on a template DNA strand. In the current study, molecular dynamics (MD) simulations are used to evaluate the accommodation of expanded/widened nucleobases in the Dpo4 active site, providing the first structural information on the replication of (x,y)DNA. Our results indicate that the Dpo4 catalytic (palm) domain is not significantly impacted by the (x,y)DNA bases. Instead, the template strand is displaced to accommodate the increased C1’–C1’ base-pair distance. The structural insights unveiled in the present work not only increase our fundamental understanding of Dpo4 replication, but also reveal the process by which Dpo4 replicates (x,y)DNA, and thereby will contribute to the optimization of high fidelity and efficient polymerases for the replication of modified nucleobases.

## 1. Introduction

At the cellular level, living organisms store and process genetic information encoded by a set of four DNA nucleobases (A, C, G, and T), which combine according to Watson-Crick hydrogen-bonding rules. There have been many attempts to expand the unique function and applications of DNA [1,2]. Approaches considered to date include editing the DNA backbone (e.g., polyamide nucleic acids (PNA), locked nucleic acids (LNA), and xeno-nucleic acids (XNA) [3,4,5]), modifying the nucleobase functional groups (e.g., difluorotoluene, isoguanine, and 2,6-diaminopurine [6,7,8,9]), or changing the underlying nucleobase composition (e.g., propynyl isocarbostyril and fleximers [10,11,12]). In addition to exploring fundamental questions about nature’s building blocks, modified DNA has many practical applications, including DNA nanomaterials [13,14], therapeutic approaches such as antiviral drugs or the delivery of genetic material to cells [15,16,17,18,19], nucleic acid computing [20,21,22,23], and tools to probe the mechanisms of biological processes [24].

**Figure 1:**
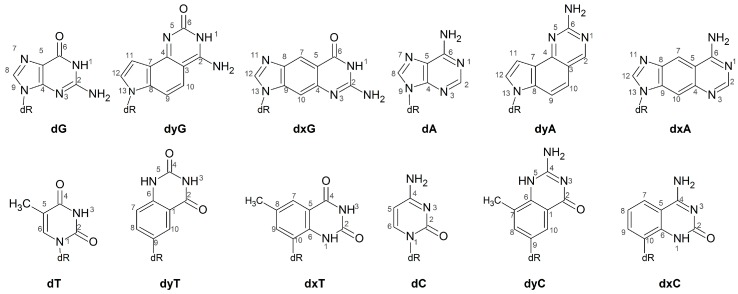
Chemical structures of the DNA, xDNA and yDNA bases considered in the current study.

One class of modified DNA that shows promising applications both as an extension of the natural genetic code and as a nucleic acid probe is expanded (x) and widened (y) DNA. These modifications have a single benzene spacer incorporated horizontally (xDNA) or vertically (yDNA) either between a natural pyrimidine base and deoxyribose, or between the 5- and 6-membered rings of a natural purine (Figure 1). Although base extension increases the base-pair C1′–C1’ distance, the canonical Watson-Crick hydrogen-bonding faces are maintained in (x,y)DNA nucleobases, which allows for complementary base pairing. The first benzene-ring expanded nucleobases were RNA analogues proposed by Leonard and coworkers [25,26,27]. The Kool group has since synthesized a series of expanded and widened DNA nucleobases [28,29,30,31,32,33,34,35]. Theoretical and experimental studies show that helices composed of (x,y)DNA:DNA base pairs are overall more stable than natural B-DNA [36,37], at least in part due to enhanced stacking [38], as well as exhibit increased charge-transfer [39,40,41] and fluorescent [36,42,43,44,45] properties. Compared to B-DNA, mixed xDNA–DNA helices have increased major and minor groove widths, and a reduced helical twist, while mixed yDNA–DNA helices have increased base-pair inclination, reduced twist, and smaller major and minor groove widths [34,35,36,41,42,46,47,48,49].

In addition to the capacity to store information through complementary pairing in stable helices, it must be possible to replicate (x,y)DNA strands in order for these modifications to be useful extensions of the natural genetic code. In biological systems, DNA is copied by either standard replication or translesion (bypass) synthesis [50]. Standard replication uses replicative DNA polymerases that have high fidelity, processivity and efficiency for canonical nucleotides, but are generally unable to replicate non-standard (e.g., damaged or otherwise modified) bases [51,52]. On the other hand, bypass polymerases exploit a highly flexible active site to replicate past many non-standard bases, albeit with lower fidelity, processivity and efficiency compared to replication of canonical DNA by standard polymerases [50]. With the goal to improve the efficiency of modified DNA replication, several research groups have focused on engineering novel polymerase variants (by modifying individual residues and/or domains) that exploit the flexibility of bypass polymerases to replicate many non-standard bases with a range of biotechnological applications [53,54,55].

Since the design of an (x,y)DNA replicase is anticipated to be extremely challenging [33], experimental studies have initially investigated the replication of (x,y)DNA by existing polymerases. This approach was at least in part fuelled by the successful replication of DNA containing expanded and widened nucleobases in *Escherichia coli (E. coli)* [56,57,58]. To gain more detailed information, kinetic studies considered the replication of a single x- or y-nucleotide (dxN or dyN) in an otherwise canonical DNA duplex by common model replicative (Klenow fragment(*exo*–), Kf(*exo*–)) and bypass (Dpo4) polymerases [30,56,59]. These studies further confirmed successful insertion of a complementary natural deoxyribose nucleoside triphosphate (dNTP) opposite d(x,y)N [30,59], but at a rate 2–3 orders of magnitude slower than complementary dNTP insertion opposite a canonical base for both polymerases [60]. Further study revealed that Kf(*exo*–) is unable to successfully extend past (x,y)DNA [30,59], whereas Dpo4 can extend past a single d(x,y)N paired with a canonical nucleobase [30,59], as well as replicate four consecutive xDNA bases [59]. Thus, replication of (x,y)DNA by Dpo4 appears to be the most promising avenue for future development considered in the literature so far. This is perhaps not surprising since Dpo4 has been shown to successfully bypass many damaged or synthetic bases, most having a large aromatic group added to a canonical nucleobase [50,61,62,63,64,65,66,67,68,69,70,71,72,73,74]. One unstudied exception is *anti*-purine:*anti*-purine mismatches, which have been speculated to impede Dpo4 nucleotide insertion by disrupting the alignment of the catalytic center because of increased C1′–C1′ distances [64]. Unfortunately, there are no studies to date that provide structural details of the influence of base size expansion on the Dpo4 active site to better appreciate how base pairs with increased C1′–C1′ distances are accommodated.

Since previous literature has demonstrated that computational studies provide critical molecular-level insight into the replication of non-standard nucleobases by Dpo4 [63,66,70,71,75,76], we have used molecular dynamics (MD) simulations to study Dpo4 replication of dxN or dyN nucleotides (Figure 1). Specifically, to align with previous experimental studies, we model a Dpo4 ternary complex for the insertion of a complementary canonical dNTP opposite an expanded (dxA, dxC, dxG, dxT), widened (dyA, dyC, dyG, dyT) or natural (dG, dT) base in an otherwise canonical DNA helix. Our simulations provide the first structural information about how the full set of expanded and widened DNA bases are accommodated in the Dpo4 active site. This data is crucial in order to better understand the process by which Dpo4 bypasses xDNA and yDNA, and thereby fully optimize a system that can replicate expanded and widened DNA with high efficiency and fidelity. Furthermore, our calculations provide the first information about structural deviations to the polymerase and DNA in the ternary (insertion) complex resulting from replication of modified bases that form pairs with increased C1′–C1′ distances. When combined with previous studies on the replication of size-increased damaged DNA nucleobases that do not form complementary Watson-Crick hydrogen bonds, our work highlights the important role of hydrogen bonding for the replication of size-extended nucleobases. Thus, the present study significantly contributes to our growing fundamental understanding of the function of Dpo4, as well as other bypass polymerases.

## 2. Results

**Figure 2:**
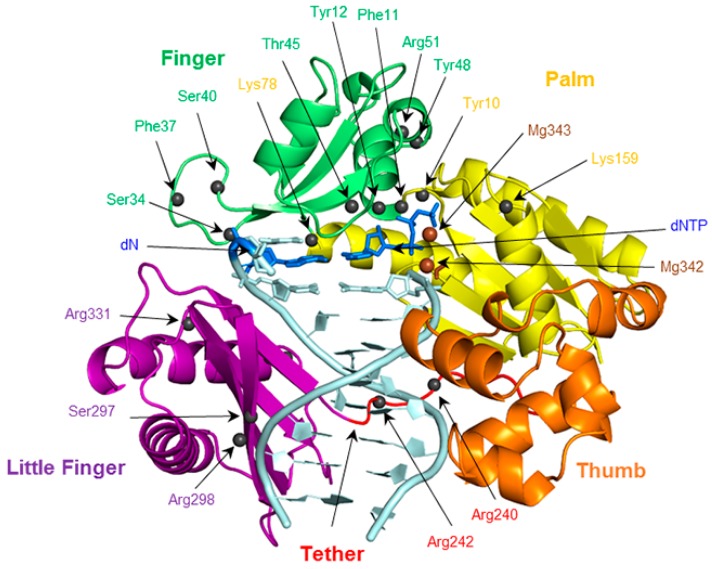
The finger, palm, thumb, and little finger domains of Dpo4, as well as the tether region. The positions of potentially important residues in each domain are indicated with spheres.

To provide the currently missing structural details of the replication of expanded and widened DNA bases, the present work systematically evaluates the conformation of the Dpo4 active site when bound to DNA containing a single (x,y)DNA base. Structural features to be considered include enzyme–dNTP interactions, the reaction parameters, d(x,y)N:dNTP hydrogen bonding, DNA structural (base-pair, base-step and helical) parameters associated with d(x,y)N:dNTP and the previously replicated base pairs, and enzyme–dN interactions. Global changes in the Dpo4 ternary complex, including characterizing any potentially significant rearrangements in each domain (palm, finger, thumb and little finger; Figure 2), as well as the tether region, are also considered. The geometrical features of the Dpo4 ternary complex for the replication of expanded or widened bases are compared with the insertion complex for natural DNA replication. Previous research indicates that canonical purine and pyrimidine dNTPs may themselves be accommodated differently in the Dpo4 active site. For example, crystal structures of the Dpo4 insertion complex indicate that stabilizing enzyme–DNA interactions vary for dCTP insertion opposite dG compared to dATP insertion opposite dT [77,78]. To account for these potential differences, the results for the replication of a modified purine or pyrimidine are compared to the corresponding Dpo4 ternary complex for the replication of either a natural purine (dG) or pyrimidine (dT), respectively. Due to the large amount of data generated, and similarities across the systems considered, the results section primarily focuses on the most significant differences between the systems.

### 2.1. Catalytic Palm Domain Does Not Significantly Rearrange for the Replication of (x,y)DNA Bases, with the Exception of Modified Cytosine

**Figure 3:**
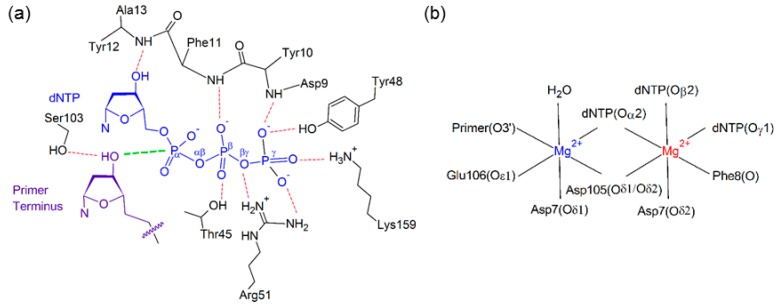
(**a**) Hydrogen-bonding interactions with the dNTP (blue) and primer terminus (purple), with the reaction distance shown in green; (**b**) Active site coordination of the catalytic (blue) and binding (red) Mg^2+^ ions.

Stabilization of the dNTP and primer strand terminus plays an important role in aligning the active center of Dpo4, which sits inside the palm domain (Figure 2). Indeed, previous computational and experimental studies have identified several interactions that position the dNTP in the Dpo4 active site (Figure 3). These include hydrogen bonds with two residues in the palm domain (Tyr10 and Lys159) and five residues in the finger domain (Thr45, Phe11, Arg51, Tyr12, and Tyr48), as well as electrostatic interactions with two Mg^2+^ ions [63,70,76,79,80,81]. Although most enzyme–dNTP interactions observed for canonical DNA replication (Figure 3a) are conserved upon base modification, there are some changes in these enzyme–dNTP interactions for the replication of (x,y)DNA nucleobases compared to the canonical counterparts, particularly for the replication of benzene-extended cytosines (Table 1 and Tables S1–S3). For example, for the replication of canonical dT, both dATP(O3′H) and Thr45(OγH) form a hydrogen bond with dATP(Oβ). However, for the replication of the modified cytosines, the corresponding hydrogen-bonding interaction with Thr45 is completely lost (Table 1), leading to a destabilization of about 8 kcal·mol^−1^ according to MM/GBSA binding energies (Table S1). Furthermore, the hydrogen bonding with Arg51 is lost due to a severely narrowed dNTP(Oβγ)···Arg51(Nη1H) hydrogen-bond angle (135° for dyC and 116° for dxC), which results in an ~10 kcal·mol^−1^ reduction in stability (Table S1). Finally, the hydrogen bonding between Tyr10(NH) and dNTP(Oγ) is reduced (occupancy decreases by ~40%; Table 1), although the associated destabilization is relatively small (~3 kcal·mol^−1^). In addition to these deviations for the modified cytosine insertion complexes, some changes occur when the replication of the modified purines are considered. For example, although there is a stabilizing interaction between the O3′ hydroxy group of dCTP and the aromatic ring of Tyr12 for the replication of dG, the O3′ hydroxy in dNTP points away from Tyr12 and towards Thr45 for the replication of dxA and dxG (Figure S1). In the dxG, dyG, dyA and dxT insertion complexes, the Lys159(NζH)···dNTP(Oγ) hydrogen-bond occupancy is reduced by ~20%, but only by 10% for dyT. Finally, the aromatic ring of Tyr48 rotates by ~45° to potentially form a stacking interaction with Arg51 when dxG or dyA are bound in the Dpo4 active site, which is not observed in the natural systems (Figure S2).

The Dpo4 active center has two hexa-coordinated Mg^2+^ ions (Figure 3b), one (catalytic Mg^2+^) that stabilizes the 3’-terminal hydroxy group of the DNA primer strand and a second (binding Mg^2+^) that stabilizes the triphosphate moiety [76,77]. Coordination of these important ions is unaffected by the modified bases, with all six coordinating bonds having greater than 99% occupancy for a distance of <2.5 Å (Table S4). We also observe a hydrogen-bonding interaction with the primer strand terminus that has not been previously reported; specifically, Ser103 hydrogen bonds with the 3′-hydroxy group of the primer strand terminus for ~90% of the simulation trajectory for the natural systems (Table S3). This occupancy decreases by 20% for dyA, and by 10% for dxC, dyC, and dxA.

### 2.2. Reaction Parameters for the Catalytic Step Are Unaffected for the Replication of All (x,y)DNA Bases

The catalytic insertion step involves water-mediated O3′ attack on Pα, formation of a pentavalent intermediate, and finally breaking of the Pα–Oαβ bond in the dNTP to free pyrophosphate from the active site [76,82]. Two important parameters that indicate correct alignment for the insertion reaction are the distance between O3′ of the primer strand terminus and the α-phosphate of the dNTP, and the angle between these atoms and Oαβ of the dNTP. The results show that the dxN and dyN bases have little effect on the alignment of the Dpo4 catalytic center (Table 2). Specifically, the average O3′–Pα distance is within 0.1 Å of the natural systems and the average O3′–Pα–Oαβ reaction angle is within 11° of the natural systems.

### 2.3. d(x,y)N:dNTP Hydrogen Bonding Does Not Significantly Deviate from Canonical Watson-Crick Interactions, with the Exception of dyA and dyC

**Figure 4:**
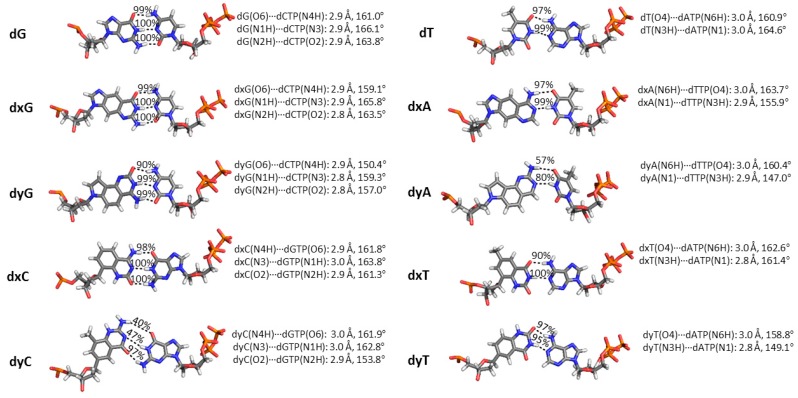
Geometry of the dN:dNTP hydrogen-bonding interaction during Dpo4 replication of a DNA, xDNA or yDNA base. Average distance and angle for each hydrogen-bonding interaction are given, as well as hydrogen-bonding occupancies, which are based on a distance cutoff of <3.4 Å and an angle cutoff of >120°.

In light of the ongoing literature debate regarding the role of hydrogen bonding in dNTP insertion by translesion polymerases [83,84,85,86], it is essential to monitor interactions between the template dN and the incoming dNTP (Figure 4). In the Dpo4 active site, dG:dCTP and dT:dATP form canonical Watson–Crick hydrogen bonds with greater than 97% occupancy. Although the Watson–Crick hydrogen-bonding pattern is maintained for all d(x,y)N:dNTP pairs, the interactions significantly change for dyA and dyC. Specifically, the average hydrogen-bond angles in the dyA:dTTP pair decrease by up to ~20° (hydrogen-bond occupancy decreases by up to 43%). For the replication of dyC, two hydrogen bonds are disrupted during the simulation (up to 60% decrease in occupancy). The next largest structural change occurs between the dyG(O6) acceptor and dCTP(N4H) donor, with the average hydrogen-bond angle narrowing by up to ~10° (10% decrease in occupancy). For the n−1 dG:dC base pair, only dxT and dyC led to a small geometric deviation from the natural systems (6% decrease in the dC(N4H)···dG(O6) hydrogen-bonding occupancy; Table S5). 

### 2.4. Base-Pair, Base-Step and Helical Parameters for (x,y)DNA Deviate from Canonical Values, with Widened Bases Generally Resulting in Greater Deviations than Expanded Bases 

**Figure 5:**
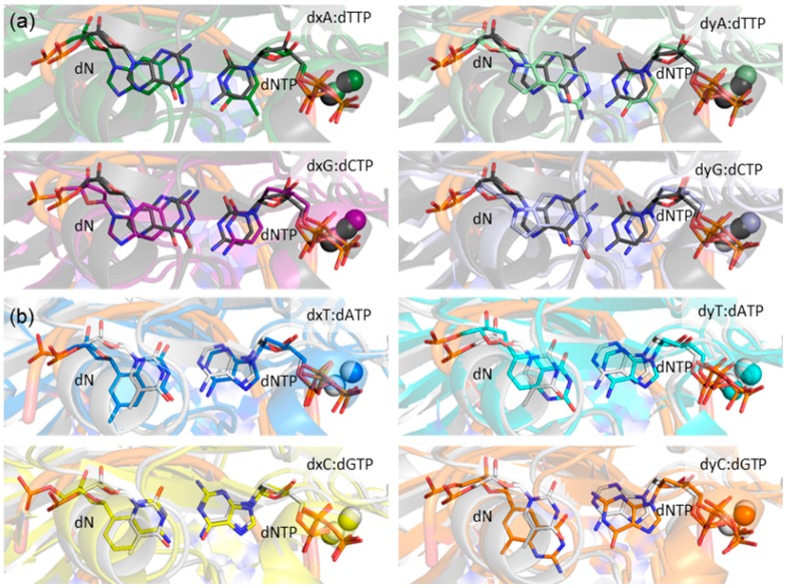
Overlays of representative MD structures of the (x,y)DNA base pairs with representative MD structures of the (**a**) dG:dCTP (black) or (**b**) dT:dATP (white) pair in the Dpo4 ternary complexes.

Expanded and widened DNA helices have been shown to adopt a native B-DNA geometry with a reduced twist, as well as increased (decreased) major and minor groove widths for xDNA (yDNA) [30]. Therefore, the geometry of the DNA helix (base-pair, base-step and helical parameters) [87,88] in the Dpo4 ternary complex for replication of an expanded or widened base was evaluated. The most significant deviation from the natural complexes in the base-pair parameters that describe the relative orientation of the d(x,y)N and dNTP bases occurs in the buckle parameter (Table 3). Specifically, dyA:dTTP, dyG:dCTP and dyT:dATP exhibit an increase in the average buckle up to ~30°, while dxA:dTTP and dxG:dCTP have an average increase in buckle of ~15°, relative to the natural systems (Figures S3 and S4). The average propeller rotation deviates by up to ~15° from the natural systems for dyA:dTTP and dyC:dGTP. The average opening of the dN:dNTP base pair is unchanged with respect to the natural complexes, with the exception of dyC:dGTP (average increases by ~14°) and dyA:dTTP (which has a large standard deviation of 12.2°). Due to the presence of the additional benzene ring in the modified bases, a large increase in stretch is observed for all complexes (Figure 5). The average stretch increases by 1.2–2.4 Å for the widened bases, but falls within a narrower range for the expanded bases (1.8–2.3 Å). Correlated to this increase in stretch, the average C1′–C1′ distance increases by ~1–2 Å compared to the natural systems. Shear displacement increases for dyA, dyG, dyT and dxC, with an average increase compare to the natural systems of up to ~2.6 Å. Finally, only the modified purines lead to a notable increase in average stagger (by up to 1.5 Å).

Among the base-step parameters that dictate the relative orientation between the dN:dNTP and n−1 dG:dC pair [87,88], only the tilt and twist are significantly affected by the presence of a modified base, and these parameters only deviate for the widened purine systems (Table 4). Specifically, the average tilt increases for dyA and dyG by up to ~10°, and the average twist for dyA is 10° larger than for natural DNA replication. The inclination and tip helical parameters are overall unchanged for the d(x,y)N base pairs with the exception of a ~12° increase in average tip for dyA:dTTP, and large standard deviations (~15°) for the inclination and tip of dxC:dGTP. Although the major groove width remains consistent across all natural and modified bases (Table 4), dyA leads to significant changes in the average minor groove width (1.6 Å decrease) and axial bend (1.7 Å increase), with associated standard deviations also being larger than for any other system. The rest of the DNA helix shows no significant changes in base-pair, base-step or helical parameters (Figures S5 and S6).

### 2.5. Canonical dN:dNTP Base-Pair Interactions Are Maintained for (x,y)DNA Bases, with the Exception of dyC, dxC and dyA

In order to appreciate the energetic effects of the base-pair and base-step distortions, the average linear interaction energies were calculated for the dN:dNTP and n−1 dC:dG pairs across the simulation trajectory (Table 5). For natural DNA replication, the dG:dCTP Watson-Crick hydrogen-bonding interaction has an electrostatic energy of –30.7 ± 2.7 kcal·mol^−1^, which is slightly higher than previously estimated using gas-phase calculations on an isolated G:C base pair (−25 to −28 kcal mol^−1^ [89]). The dT:dATP interaction is −7.1 ± 1.6 kcal·mol^−1^, which is ~5 kcal mol^−1^ lower than previously estimated for an isolated A:T base pair (−12 kcal·mol^−1^ [89]). The hydrogen-bond interaction in the n−1 dG:dC pair maintains the strength of a canonical pair, ranging between −27.6 and −28.8 kcal·mol^−1^ regardless of the base being replicated. Although the majority of the (x,y)DNA pairs maintain the stability of their canonical counterparts, dyC:dGTP is significantly less stable, with a hydrogen-bond energy of only −4.5 ± 2.9 kcal·mol^−1^, as expected due to changes in the base pair geometry. The only other significant differences are the reduced hydrogen-bonding energy for dxC:dGTP (−23.4 ± 2.6 kcal·mol^−1^) and increased standard deviation for dyA:dTTP (−5.8 ± 3.5 kcal·mol^−1^).

The stacking interactions between the d(x,y)N:dNTP and n−1 dC:dG pairs reported herein (Table 5) are within 1–2 kcal·mol^−1^ of the empirical (AMBER) van der Waals energies of isolated stacked base pairs [90]. Indeed, the stacking energies for the expanded bases are not significantly greater than for the natural bases, and the stacking energies for the expanded and widened bases are consistently within 1 kcal·mol^−1^. The (x,y)purine stacking energies with the n−1 dC are on average 2 kcal·mol^−1^ more stabilizing than the analogous (x,y)pyrimidine interactions. Interestingly, dxT and dyC have stabilizing stacking interactions with the n+1 dC, largely attributed to an interaction between the methyl group in dxT or dyC and the n+1 dC (Figure S7). This methyl interaction anchors the n+1 dC above the modified base, which inhibits the n+1 dC from rotating away from the reaction center as previously reported in crystal structures of natural and damaged DNA [91], and observed for all other modified bases in the present work.

### 2.6. Residues Interacting with the Template Backbone Move to Accommodate the (x,y)DNA Bases

**Figure 6:**
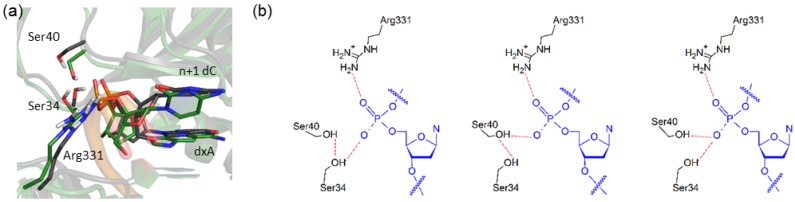
Alterations in the Dpo4 conformation to accommodate an (x,y)DNA base in the template strand. (**a**) Overlay of the representative MD structures of the Dpo4 ternary complex for the replication of dxA (green) and dG (black). Overlays for all systems are provided in the Supplementary Material (Figure S8); (**b**) Possible hydrogen-bonding arrangements within the Ser40···Ser34···d(x,y)N chain.

The largest structural deviations in the Dpo4 ternary complexes for (x,y)DNA versus canonical DNA replication occur near the expanded or widened base. Specifically, the extended base size causes the backbone of the template strand to shift outward (i.e., in the direction of an increased stretch parameter, see Figure 5). This shift is on average ~2.5 Å for the expanded and widened pyrimidines, except for dxC where the backbone shifts on average 3.0 Å (Figure 5 and Table 6). The expanded and widened purines lead to smaller average movement of 1.2–1.8 Å. Regardless, this motion directly impacts stabilizing interactions between the protein and the backbone of the template strand, particularly contacts involving Lys78, Arg331, Ser34, and Ser40. Specifically, in the natural complexes, there is a stabilizing interaction (3–7 kcal·mol^−1^; Table S6) between Lys78 (palm domain) and the template backbone at the n−2 position that is present for up to 53% of the simulations (Table S7). This interaction is lost in all modified systems. Arg331 forms a hydrogen-bonding interaction with the backbone of the n+1 dC for natural DNA replication (Figure 6a and Table S8). Although this interaction is still present for dxN or dyN replication, the increase in base-pair size shifts Arg331, causing the α-carbon of the protein backbone to move outwards by up to an average distance of 1.4 Å for the modified systems (Figure S8). Finally, in the natural systems, the hydroxy groups of Ser34 and Ser40 form a cooperative hydrogen-bond chain with the dN phosphate moiety (i.e., Ser40(OγH)···Ser34(OγH)···dN(OP); Figure 6b, left). Although this hydrogen-bonding pattern is maintained for dxC replication, the hydrogen-bonding chain switches direction in the dyG complex (Figure 6b, middle). In contrast, Ser40 is directed away from the helix backbone and forms a Phe37(NH)···Ser40(Oγ) hydrogen bond for the majority of the dxG simulation. For all other (x,y)DNA bases, both Ser34 and Ser40 form direct hydrogen bonds with the same d(x,y)N(OP) atom (Figure 6b, right).

### 2.7. Residues in the Ceiling Region of the Finger Domain Rearrange to Accommodate the (x,y)DNA Bases

Alignment of the ceiling region of the finger domain (Val32, Ala42, Ala44, Asn47, Gly58, and Met76) that spans the dN:dNTP base pair is important for replication, and may be one factor that prevents base-pair mismatches [64,77,85]. For (x,y)DNA replication, the ceiling residues undergo some movement relative to the natural complexes (Figure S9). Specifically, for all (x,y)DNA bases, Met76 shifts away from the dN:dNTP pair by on average 0.7–0.9 Å (Table 6). Additionally, Val32 rotates towards dN:dNTP for dxG, dyG, dxT and dxC. Gly58 moves towards the dN:dNTP by ~1.0–1.3 Å for dyC, dxT and dxG, stabilized by a hydrogen bond between the template n+1 dC amine group and the Gly58 backbone carboxylate. For replication of the remaining modified bases, Gly58 moves away from the dN:dNTP pair by on average 0.8–1.2 Å. 

### 2.8. Accomodation of the Modified Bases Disrupts the Enzyme Backbone, Particularly for the Finger and Little Finger Domains

**Figure 7:**
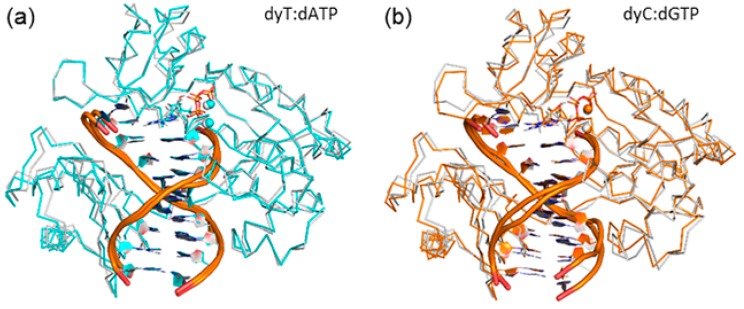
Overlay of the Cα protein backbone in representative MD structures of the Dpo4 ternary complex for the replication of: (**a**) dyT (blue); or (**b**) dxC (yellow), and dT (white). See Figure S10 for all systems.

To determine whether accommodation of d(x,y)N causes any significant changes in the overall Dpo4 ternary complex, beyond displacement of the template strand and the changes to the Dpo4:DNA hydrogen-bonding contacts previously discussed, the global movements of each domain were systematically analyzed. The RMSD per domain over each simulation (calculated relative to the corresponding representative structure) indicates that each domain exhibits nearly the same stability throughout the trajectory (Table S9). When the global structure of the d(x,y)N complexes are compared to the natural complexes, a repositioning of the protein backbone of the finger, thumb and little finger domains, as well as the tether region is observed and reemphasizes the structural conservation of the palm domain (Figure 7 and Figure S10). Furthermore, the smallest deviations from the natural systems occur for the dyT complex (Figure 7a), while the largest deviations occur for dxC (Figure 7b). These deviations are present throughout the simulation trajectories (Table S10), albeit slightly diminished when the complete modified simulation trajectories rather than single representative structures are compared to the corresponding natural representative structure. Despite changes in the enzyme–DNA hydrogen-bonding contacts in the finger region previously discussed, the observed repositioning does not significantly affect enzyme–DNA interactions in any other domain (Tables S3, S7, S8, and S11–S15).

## 3. Discussion

Our molecular dynamics simulations provide important structural information for the experimentally observed replication of expanded (x) and widened (y) DNA nucleobases by the model bypass polymerase (Dpo4). Since previous literature proposed that Dpo4 replication of DNA containing base pairs with increased C1′–C1′ distances may disrupt the alignment of the catalytic center [64], we initially focused on the structure of the active site relative to canonical DNA replication. The efficiency of the catalytic step is known to depend on discrete interactions between several Dpo4 residues and the dNTP backbone, as well as the coordination of two Mg^2+^ ions [76]. Although the coordination of the Mg^2+^ ions was unchanged relative to the natural complexes and the majority of enzyme–DNA hydrogen-bonding interactions are maintained, some significant differences in the interactions with the active site residues were observed. For example, the dNTP hydrogen-bonding interactions with Arg51, Thr45 and Tyr10 were disrupted for the replication of dxC and dyC, while Lys159 shifted in the ternary replication complexes for dyA, dxG, dyG, dxT and dyT. However, the changes observed for active site residues relative to natural DNA replication were not consistent for all modified bases. Another factor that may influence replication is the geometry of the newly forming base pair. Although the Watson-Crick hydrogen-bonding pattern was maintained for all modified bases, the geometry of the pair significantly fluctuates for dyA and dyC, which decreases the canonical interaction energy by up to 25 kcal·mol^−1^. Furthermore, while the overall base-step parameters are not considerably altered, there is a significant deviation in at least one base-pair parameter for each modified base (particularly buckle, propeller, opening or stretch), with the deviations typically being larger for the widened bases. Regardless of these differences, the reaction (distance and angle) parameters for dNTP insertion opposite expanded and widened bases are unchanged relative to natural DNA replication. Conservation of these reaction parameters correlates with the experimentally observed ability of Dpo4 to replicate (x,y)DNA [30,59]. Furthermore, some of the disruptions observed relative to the natural systems in our calculations may at least in part explain the up to 3,600 times less efficient replication of (x,y)DNA compared to the replication of natural DNA [30,59]. Additionally, there are notable correlations between trends in the experimentally observed reduction in replication rates and MD structural information for the ternary complex. Specifically, among the widened bases, dyT causes the least structural distortion to the insertion complex and correspondingly has the smallest reduction in the experimentally observed replication rate among the yDNA bases [59]. In contrast, the dxC insertion complex has the largest calculated distortion among the expanded bases, which correlates with the greatest reduction in replication rate among the xDNA bases [30]. Nevertheless, not all experimental observations for the replication of d(x,y)N can be correlated with the results obtained from MD simulations of the Dpo4 insertion complex. Indeed many factors govern the experimentally observed decrease in rate, such as reaction barriers, pyrophosphate departure and/or pre/post-catalytic conformational changes, which cannot be accounted for in simulations of ternary replication complexes.

In order to maintain both the geometry of the active site and Watson–Crick hydrogen bonding, while accommodating a dN:dNTP pair with an increased C1′–C1′ distance, Dpo4 allows the template backbone to shift away from the reaction center by up to 3 Å. This movement has direct impact on contacts between the template strand and Dpo4, including interactions with Lys78, Arg331, Ser34 and Ser40. Furthermore, interactions between the dN:dNTP pair and the ceiling region are affected by the accommodation of the (x,y)DNA base, particularly those involving Val32, Gly58 and Met76. Indeed, when representative structures of Dpo4 bound to DNA helices containing the modified bases are compared to those for the canonical counterparts (Figure 7 and Figure S10), the largest enzyme deviations are seen in the (finger and little finger) domains that surround the template strand, while there are relatively small deviations in the (palm) domain that contains the catalytic center. This reveals that it is the flexibility of the finger and little finger domains that allows Dpo4 to accommodate size-extended bases, without compromising the interactions required to stabilize and position the dNTP for catalytic nucleotide insertion. This flexibility may also explain the experimentally observed reduction in fidelity for Dpo4 replication of expanded bases, as well as the ability of Dpo4 to extend past (x,y)DNA, which was not possible for the more rigid Kf(*exo*–) polymerase [30,56,59]. Our conclusions correlate with previous computational studies of Dpo4 preinsertion, insertion and extension complexes for natural DNA replication, which conjecture that the finger and little finger domains are the most flexible regions and therefore may aid accommodation of modified DNA [81].

**Figure 8:**
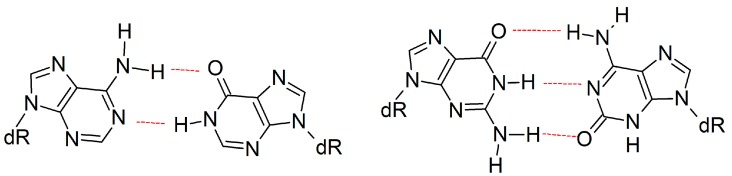
Examples of size-extended *anti*-purine:*anti*-purine mismatches.

The present work can be used to gain insight into the replication of other modified bases that form complementary hydrogen-bonded base pairs with increased C1′–C1′ distances. For example, *anti*-purine:*anti*-purine mismatches (e.g., adenine:hypoxanthine and guanine:isoguanine; Figure 8) are of great interest due to their relation to the origin of life and developing an understanding of why genetic information is stored in specific purine:pyrimidine base pairs [92,93,94,95,96]. Although *anti*-purine:*anti*-purine mismatches have been shown to form DNA helices with a stability that is similar to analogous purine:pyrimidine helices [92,93,94], the replication of such purine:purine pairs has not been studied to the best of our knowledge. Nevertheless, it has been speculated that the increased C1′–C1′ distance will impede replication by misaligning the Dpo4 active center, particularly in the area surrounding the catalytic Mg^2+^ ions [64]. However, this proposal directly contrasts our current observations for the replication of an expanded or widened DNA base. Therefore, we instead propose that movements in the finger and little finger domains of Dpo4 will accommodate DNA containing *anti*-purine:*anti*-purine mismatches, while maintaining the active center conformation and allowing for replication.

**Figure 9:**
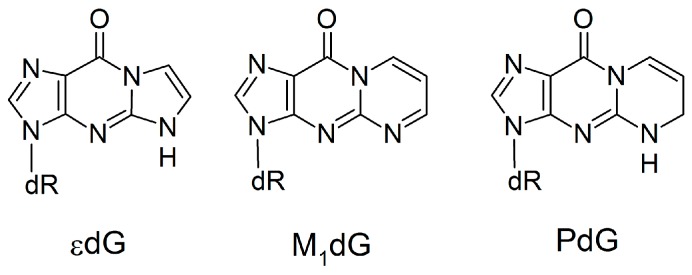
Damaged DNA bases with extended ring systems that lack Watson–Crick hydrogen bonding.

Unlike *anti*-purine:*anti*-purine mismatches, experimental studies have investigated the Dpo4 replication of damaged DNA bases that would lead to pairs with increased C1’–C1’ distances, such as 1,N^2^-ethenoguanine (εdG), malondialdehyde-guanine (M_1_dG) and 1,N^2^-propanoguanine (PdG; Figure 9) [97,98,99]. Similar to the (x,y)DNA bases, these damaged bases have expanded ring systems; however, the damaged bases lack the Watson-Crick hydrogen-bond face of dG. According to published crystal structures [97,98,99], these lesions do not significantly change the overall enzyme structure. However, the incoming dNTP is not accommodated opposite the expanded bases, which instead result in 1-base deletion mutations. Since 1-base deletion mutations are formed because the dNTP pairs opposite the n+1 base, the reaction parameters for modified base replication are misaligned and therefore there is a low replication efficiency (up to 2 × 10^4^ times slower than canonical DNA replication) [97,98,99]. This contrasts the observed lack of deletion mutations and successful replication of (x,y)DNA [30,59], which our work reveals maintains canonical Watson-Crick pairing within the Dpo4 active site through movement of the template DNA strand and surrounding enzyme domains. Comparison of these two general classes of modified DNA highlights the important role that hydrogen bonding plays in the replication of size-extended bases by Dpo4. Specifically, dN:dNTP hydrogen bonding can act as a driving force to move the template strand rather than allowing size-extended bases to disrupt the active site configuration. Interestingly, this conclusion contrasts previous assertions for the replication of modified bases that form pairs with the same shape (and therefore size) as canonical pairs, which do not have a strict requirement of dN:dNTP hydrogen bonding for nucleotide insertion [85,86,100,101]. 

## 4. Materials and Methods 

Starting structures for the MD simulations were obtained from a crystal structure of the Dpo4 ternary complex for the insertion of dATP opposite dT during extension past the benzo[a]pyrene adenine adduct (PDB ID: 1S0M, chains B, E and F) [102]. The catalytic divalent ions (Ca403 and Ca404) were modified to Mg^2+^ ions. Two excess divalent ions (Ca405 and Ca408) are crystallization artifacts and were removed. The template DNA strand sequence was modified to 5′–CNCCATCGCC (Figure S11), with dxN (dxA, dxC, dxG, or dxT) or dyN (dyA, dyC, dyG, or dyT; Figure 1) positioned at N. A complementary 8-mer primer DNA strand was paired opposite the 10-mer template strand and a complementary canonical dNTP nucleotide paired opposite dN. The inclusion of a single modified base in a canonical helix is consistent with previous experimental studies of the Dpo4 replication of (x,y)DNA [30,59]. To account for any differences in the polymerase active site upon purine or pyrimidine replication [77,78], two control simulations were performed by placing dG or dT at N paired opposite dCTP or dATP, respectively. The resulting 10 complexes were prepared for minimization using the tleap module of AmberTools 14 [103]. Specifically, hydrogen atoms were added to generate the natural protonation states of all DNA and protein residues, and the systems were neutralized with 5 Na^+^ ions and solvated in an octahedral TIP3P water box such that the solute is at least 12.0 Å from the edge of the box. The resulting complexes each contain 341 amino acids, a dNTP, 18 nucleotides, 2 Mg^2+^ ions, 5 Na^+^ ions and ~15,300 water molecules. 

The natural amino acids, nucleotides, and solvent were modeled using AMBER ff14SB parameters [104]. Ions were modeled using the 12-6-4 Lennard Jones-type non-bonded parameters of Li and Merz [105]. The dNTP, dxN and dyN parameters were assigned according to the GAFF [106] and AMBER ff14SB [104] force fields using the antechamber module of Amber 14 [103]. dNTP charges were taken from the literature [70,107,108]. Partial charges for dxN and dyN (Supplementary Tables S16–S23) were calculated using Gaussian 09 [109] (B3LYP/6-31+G(d)) and determined using RESP charge fitting with the PyRED (SEP-2015) server [109,110,111,112,113]. 

For all systems, the solvent was initially minimized and equilibrated using 1000 steps of steepest decent and 1000 steps of conjugate gradient minimization, followed by 10 ps of constant energy equilibration using a 0.001 ps time step. During solvent minimization and equilibration, a 50 kcal·mol^−1^·Å^−2^ force constraint was placed on the protein, DNA and ions. Subsequently, the same steps were performed to minimize the solvent and Na^+^ ions by placing a 50 kcal·mol^−1^·Å^−2^ force constraint on the protein, DNA and Mg^2+^ ions. Next, the Dpo4 complex was minimized using 5000 steps of steepest decent, followed by 5000 steps of conjugate gradient minimization, with a 25 kcal·mol^−1^·Å^−2^ force constraint on the solvent and Na^+^ ions. The solvent and Na^+^ ions were then re-minimized using 100 steps of steepest decent, followed by 500 steps of conjugate gradient minimization, with a 25 kcal·mol^−1^·Å^−2^ force constraint on the protein, DNA and Mg^2+^ ions. A non-bonded cutoff of 8.0 Å was implemented in all initial minimization and equilibration calculations.

Following initial minimization and equilibration calculations, the system was heated from 10.0 to 310.0 K over 10 ps using a 0.001 ps time step and the weak-coupling algorithm with a time constant of 2.0 ps, and a 25 kcal·mol^−1^·Å^−2^ force constraint on the protein, DNA and Mg^2+^ ions. To ensure the systems reached the desired temperature of 310.0 K, the heating step was repeated, this time heating the system from 275.0 to 310.0 K. Next, the force constraints on the protein, DNA and Mg^2+^ ions were slowly lifted while maintaining the temperature at 310.0 K using Langevin dynamics (collision frequency of 3.0 ps^−1^). Specifically, the restraints were lifted over four steps, with the restraints set to 20, 5, 2.5 and 1.5 kcal·mol^−1^·Å^−2^, and run for 10, 30, 20, and 20 ps in each step, respectively. These calculations invoked a time step of 0.002 ps, the SHAKE algorithm and periodic boundary conditions, and maintained the pressure of the system at 1.0 bar via isotropic position scaling (pressure relation time of 1.0 ps). In the final equilibration, 2 ns of unrestrained simulation were performed with a 0.002 ps time step, SHAKE and periodic boundary conditions, the temperature maintained at 310.0 K through Langevin dynamics (collision frequency of 3.0 ps^−1^), the pressure maintained at 1.0 bar through isotropic position scaling (pressure relation time of 1.0 ps), and a non-bonded cutoff of 10.0 Å implemented. 

Following minimization and equilibration, a 40 ns unrestrained production MD simulation with a 0.002 ps time step was performed on each of the 10 systems. During the production simulation, isotropic position scaling (pressure relation time of 2.0 ps) was used to maintain the pressure of the system at 1.0 bar, and the temperature of the system was held constant at 310.0 K through Langevin dynamics (collision frequency of 3.0 ps^−1^). SHAKE and periodic boundary conditions were implemented for production runs, with a non-bonded cutoff of 10.0 Å. Simulations were performed using the pmemd module of Amber 14 [103]. Each simulation remained stable, with a backbone RMSD of 1.1–1.6 Å over the 40 ns simulation (Table S24).

Base-step parameters were evaluated using Curves+ [114], while all other structural and energetic analyses was completed using AmberTools 14 [103]. For the energetic analysis of enzyme–DNA interactions, Molecular Mechanics/Generalized Born Surface Area (MM/GBSA) pairwise energies were calculated for binding of the helix (including dNTP) to Dpo4. Furthermore, in line with previous reports [90], linear interaction energies were used to evaluate dN:dNTP hydrogen-bonding (electrostatic component) and stacking energies (van der Waals component) energies. 

## 5. Conclusions

We have provided the first structural information for the replication of an expanded or widened DNA base by Dpo4. Our calculations reveal that an (x,y)DNA base paired opposite a canonical dNTP maintains Watson-Crick hydrogen bonding within the Dpo4 active site, albeit with some deviations to the base-pair and base-step parameters. The Dpo4 active site accommodates the increased C1′–C1′ distance in these base pairs by allowing the template DNA strand to displace relative to the strand position during the replication of a natural base. As a result, the majority of hydrogen-bonding interactions between the enzyme and the incoming dNTP are maintained, while some deviations in the hydrogen-bonding contacts with the template strand occur. This allows the reaction parameters to remain well aligned for dNTP insertion, which correlates with the experimentally observed successful replication of (x,y)DNA bases [30,59]. Since we did not observe consistent changes in the modified ternary complexes across the expanded and widened bases, further studies are required to fully explain the experimentally observed low Dpo4 replication efficiency for the (x,y)DNA bases [30,59]. 

In addition to providing insight into how expanded and widened DNA bases are accommodated in the Dpo4 active site, this work increases our fundamental understanding of Dpo4 function. First, although it was previously proposed that *anti*-purine:*anti*-purine mismatches with complementary hydrogen bonding and increased C1′–C1′ distances would misalign the catalytic center and thus hinder replication, our work suggests that size-extended base pairs can be accommodated by moving the template strand and associated Dpo4 domains rather than disrupting the reaction site. Second, our work highlights that dN:dNTP hydrogen bonding plays a critical role in the bypass of size-extended bases that do not have the same shape as the canonical counterparts. Although these proposals must be validated by future experimental studies investigating the replication of a range of modified nucleobases that result in size-extended base pairs, our work contributes to the growing body of literature geared towards understanding the function of Dpo4 and other bypass polymerases.

## Figures and Tables

**Table 1:** Percent occupancies for hydrogen-bonding interactions between the dNTP and active site residues ^a^.

Active Site Base Pair	dNTP(Oβγ) Arg51(Nη1H)	dNTP(Oγ) Arg51(Nη2H)	dNTP(Oγ) Lys159(NζH)	dNTP(Oγ) Tyr10(NH)	dNTP(Oβ) Thr45(OγH)	dNTP(Oβ) dNTP(O3′H)
dG:dCTP	100%	97%	90%	98%	99%	8%
dT:dATP	98%	99%	88%	99%	96%	99%
dxA:dTTP	98%	100%	93%	96%	98%	11%
dyA:dTTP	93%	100%	76%	98%	100%	NA ^b^
dxG:dCTP	100%	90%	68%	98%	97%	6%
dyG:dCTP	99%	90%	71%	98%	95%	NA ^b^
dxT:dATP	99%	97%	61%	99%	96%	100%
dyT:dATP	99%	98%	78%	99%	96%	100%
dxC:dGTP	NA ^b^	100%	99%	44%	NA ^b^	93%
dyC:dGTP	NA ^b^	100%	99%	41%	NA ^b^	93%

^a^ Hydrogen-bonding occupancies are based on a distance cutoff of <3.4 Å and an angle cutoff of >120°. Table S2 also contains average distances and angles of hydrogen-bonding interactions with the dNTP; ^b^ Not observed with >5% occupancy within our hydrogen-bonding criteria.

**Table 2:** Average and standard deviation of the reaction distance and angle during 40 ns MD simulations of the Dpo4 ternary complex for the replication of a DNA, xDNA or yDNA base.

Active Site Base Pair	d(O3′–Pα)	∠(O3′–Pα–Oαβ)
dG:dCTP	3.6 ± 0.1 Å	172.9 ± 3.7°
dT:dATP	3.5 ± 0.1 Å	174.0 ± 3.0°
dxA:dTTP	3.6 ± 0.1 Å	174.4 ± 3.0°
dyA:dTTP	3.6 ± 0.2 Å	174.2 ± 3.1°
dxG:dCTP	3.6 ± 0.1 Å	174.1 ± 3.2°
dyG:dCTP	3.6 ± 0.1 Å	174.2 ± 3.1°
dxT:dATP	3.5 ± 0.1 Å	174.1 ± 3.1°
dyT:dATP	3.5 ± 0.1 Å	173.7 ± 3.3°
dxC:dGTP	3.6 ± 0.2 Å	163.1 ± 4.5°
dyC:dGTP	3.6 ± 0.2 Å	162.6 ± 4.5°

**Table 3:** Averages and standard deviations of the base-pair parameters during 40 ns MD simulations of the Dpo4 ternary complex for the replication of a DNA, xDNA or yDNA base ^a,b^.

Active Site Base Pair	Buckle	Propeller	Opening	Shear	Stretch	C1′–C1′ Distance	Stagger
dG:dCTP	–5.3 ± 7.4	–1.2 ± 7.1	0.8 ± 2.9	0.2 ± 0.3	0.0 ± 0.1	10.8 ± 0.1	–0.3 ± 0.4
dT:dATP	1.5 ± 7.8	–4.7 ± 7.1	0.6 ± 4.7	–0.1 ± 0.3	0.1 ± 0.1	10.8 ± 0.2	–0.1 ± 0.4
dxA:dTTP	–14.2 ± 9.3	–9.5 ± 7.1	5.5 ± 5.0	0.4 ± 0.3	2.2 ± 0.2	12.6 ± 0.2	–1.2 ± 0.7
dyA:dTTP	–28.7 ± 13.6	–19.8 ± 9.5	3.7 ± 12.2	–2.6 ± 0.6	1.2 ± 0.3	11.9 ± 0.5	–1.9 ± 0.9
dxG:dCTP	–18.2 ± 9.6	–6.0 ± 7.2	1.5 ± 3.5	0.4 ± 0.3	2.3 ± 0.1	12.8 ± 0.2	–1.3 ± 0.6
dyG:dCTP	–30.6 ± 10.2	–10.1 ± 7.9	–3.3 ± 3.5	–1.3 ± 0.4	1.7 ± 0.1	12.4 ± 0.2	–1.7 ± 0.7
dxT:dATP	–8.5 ± 7.8	–7.2 ± 9.3	1.3 ± 5.9	0.4 ± 0.3	1.8 ± 0.2	12.2 ± 0.2	–0.4 ± 0.5
dyT:dATP	–24.2 ± 13.1	–2.7 ± 7.5	5.1 ± 3.7	–1.2 ± 0.3	1.9 ± 0.2	12.2 ± 0.3	–0.1 ± 0.7
dxC:dGTP	3.3 ± 6.8	–6.3 ± 9.1	3.1 ± 3.8	1.2 ± 0.3	2.1 ± 0.1	12.7 ± 0.2	0.3 ± 0.4
dyC:dGTP	0.1 ± 9.3	–15.1 ± 8.5	13.8 ± 14.0	–0.3 ± 0.9	2.4 ± 0.4	12.4 ± 0.5	–0.2 ± 0.4

^a^ See Figure S5 for the base-pair parameters for the rest of the helix; ^b^ Buckle, propeller and opening are in degrees, while shear, stretch, C1′–C1′ distance and stagger are in Angstroms.

**Table 4:** Averages and standard deviations of the base-step and helical parameters during 40 ns MD simulations of the Dpo4 ternary complex for the replication of a DNA, xDNA or yDNA base ^a,b^.

Active Site Base Pair	Tilt	Roll	Twist	Shift	Slide	Rise	Inclination	Tip	Major Groove Width	Minor Groove Width	Axial Bend
dG:dCTP	–4.1 ± 3.5	7.7 ± 4.1	26.3 ± 3.1	0.2 ± 0.4	–0.8 ± 0.3	3.2 ± 0.2	18.2 ± 4.5	0.1 ± 4.4	14.0 ± 1.4	8.3 ± 1.8	2.2 ± 1.0
dT:dATP	–0.7 ± 4.0	5.0 ± 4.7	29.8 ± 3.1	0.4 ± 0.5	–0.9 ± 0.3	3.2 ± 0.3	14.0 ± 5.7	0.1 ± 4.2	13.4 ± 1.6	8.0 ± 2.0	2.0 ± 0.9
dxA:dTTP	–7.7 ± 4.2	4.9 ± 3.8	21.9 ± 2.8	0.1 ± 0.5	–1.2 ± 0.3	3.1 ± 0.2	17.8 ± 5.7	4.6 ± 4.5	13.2 ± 0.9	7.8 ± 1.7	2.2 ± 1.1
dyA:dTTP	–17.3 ± 7.5	4.8 ± 4.6	36.1 ± 4.0	0.0 ± 0.7	0.0 ± 0.8	2.9 ± 0.3	23.3 ± 6.5	12.4 ± 5.5	13.5 ± 1.0	6.4 ± 3.7	3.7 ± 2.0
dxG:dCTP	–8.0 ± 4.3	6.2 ± 4.1	22.1 ± 3.0	–0.1 ± 0.4	–1.2 ± 0.3	3.1 ± 0.2	20.5 ± 5.3	3.9 ± 4.6	13.3 ± 1.0	7.6 ± 2.7	3.2 ± 1.5
dyG:dCTP	–12.4 ± 5.2	4.6 ± 3.7	28.2 ± 3.0	–0.1 ± 0.4	–0.6 ± 0.4	3.0 ± 0.3	20.2 ± 4.8	8.6 ± 4.6	13.2 ± 1.1	8.5 ± 1.9	2.1 ± 1.1
dxT:dATP	–1.8 ± 4.0	5.8 ± 4.4	25.1 ± 3.2	0.3 ± 0.5	–1.4 ± 0.3	3.1 ± 0.2	15.0 ± 5.4	–1.1 ± 4.3	13.5 ± 1.0	8.1 ± 1.3	2.7 ± 1.2
dyT:dATP	–4.4 ± 4.8	1.4 ± 5.2	29.9 ± 3.0	–0.2 ± 0.4	–0.9 ± 0.4	3.3 ± 0.3	12.0 ± 5.3	4.1 ± 5.0	13.3 ± 1.2	8.2 ± 1.4	2.5 ± 1.2
dxC:dGTP	–5.8 ± 3.3	5.7 ± 4.1	19.8 ± 3.4	0.2 ± 0.4	–1.5 ± 0.3	3.4 ± 0.2	19.7 ± 14.6	–1.9 ± 14.7	13.1 ± 0.9	7.9 ± 1.1	2.6 ± 1.1
dyC:dGTP	–6.8 ± 3.8	10.0 ± 5.0	26.5 ± 3.5	–0.6 ± 0.8	–0.9 ± 0.4	3.3 ± 0.2	22.0 ± 7.0	0.9 ± 4.6	13.6 ± 1.2	8.4 ± 2.0	1.9 ± 1.0

^a^ See Figure S6 for the base-step parameters for the rest of the helix; ^b^ Tilt, roll, twist, incination, tip and axial bend are in degrees, while shift, slide, rise and major/minor groove widths are in Angstroms.

**Table 5:** AMBER linear interaction energies (kcal·mol^−1^) for the dN:dNTP base pair during 40 ns MD simulations of the Dpo4 ternary complex for the replication of a DNA, xDNA or yDNA base ^a^.

Active Site Base Pair	dN:dNTP Hydrogen Bonding	dC:dG n−1 Hydrogen Bonding	dNTP–dG n−1 Stacking	dN–dC n−1 Stacking	dN–dC n+1 Stacking	dN:dNTP–dC:dG n−1 Stacking
dG:dCTP	−30.7 ± 2.7	−29.3 ± 2.5	−6.1 ± 0.6	−6.6 ± 0.6	−0.3 ± 0.2	−15.4 ± 1.0
dT:dATP	−7.0 ± 1.6	−28.8 ± 2.7	−7.1 ± 0.6	−5.1 ± 0.7	−0.4 ± 0.2	−15.1 ± 0.9
dxA:dTTP	−9.0 ± 2.0	−27.9 ± 3.0	−6.6 ± 0.6	−7.3 ± 0.7	−0.4 ± 0.3	−16.8 ± 1.0
dyA:dTTP	−5.8 ± 3.5	−27.6 ± 3.0	−5.5 ± 0.9	−6.9 ± 0.7	−0.4 ± 0.4	−15.6 ± 1.3
dxG:dCTP	−26.9 ± 2.7	−28.3 ± 2.8	−6.0 ± 0.6	−7.6 ± 0.7	−1.5 ± 1.1	−16.8 ± 1.0
dyG:dCTP	−31.4 ± 3.8	−28.0 ± 2.7	−5.7 ± 0.6	−6.9 ± 0.7	−0.5 ± 0.4	−15.7 ± 1.0
dxT:dATP	−9.4 ± 1.6	−27.6 ± 3.2	−6.9 ± 0.7	−4.6 ± 0.7	−1.9 ± 1.4	−14.0 ± 1.1
dyT:dATP	−9.3 ± 1.7	−27.6 ± 3.0	−6.9 ± 0.8	−4.1 ± 0.8	−0.6 ± 0.7	−13.4 ± 1.2
dxC:dGTP	−23.4 ± 2.6	−27.8 ± 3.0	−7.2 ± 0.7	−5.5 ± 0.7	0.0 ± 0.1	−15.6 ± 1.1
dyC:dGTP	−4.5 ± 2.9	−27.9 ± 2.8	−7.6 ± 0.8	−5.4 ± 0.9	−3.3 ± 1.7	−14.4 ± 1.3

^a^ The hydrogen-bonding energies are reported as the electrostatic component, while the stacking interactions are reported as the van der Waals energy component.

**Table 6:** RMSD (Å) in the backbone position in the Dpo4 ternary complex for the replication of a (x,y)DNA base compared to a natural base across the 40 ns MD simulation trajectories ^a^.

Active Site Base Pair	dN	Template n−1	dNTP	Primer n−1	Ser34	Ser40	Gly58	Met76	Arg331
dxA:dTTP	1.7 ± 0.5	1.0 ± 0.3	0.5 ± 0.1	0.7 ± 0.5	1.1 ± 0.4	1.1 ± 0.4	0.8 ± 0.4	0.7 ± 0.3	1.4 ± 0.4
dyA:dTTP	1.2 ± 0.4	0.7 ± 0.3	0.4 ± 0.1	0.8 ± 0.5	1.1 ± 0.4	1.0 ± 0.4	0.9 ± 0.4	0.7 ± 0.3	0.8 ± 0.4
dxG:dCTP	1.8 ± 0.4	1.0 ± 0.3	0.5 ± 0.1	0.7 ± 0.4	1.1 ± 0.4	0.9 ± 0.5	0.9 ± 0.4	0.7 ± 0.3	1.1 ± 0.4
dyG:dCTP	1.4 ± 0.3	0.9 ± 0.3	0.5 ± 0.2	0.8 ± 0.3	1.1 ± 0.4	1.1 ± 0.4	0.8 ± 0.4	0.7 ± 0.3	1.2 ± 0.4
dxT:dATP	3.0 ± 0.8	1.4 ± 0.4	0.4 ± 0.2	0.8 ± 0.4	0.9 ± 0.4	1.0 ± 0.5	1.0 ± 0.4	0.7 ± 0.3	1.3 ± 0.4
dyT:dATP	2.6 ± 1.0	1.0 ± 0.4	0.5 ± 0.2	0.8 ± 0.4	1.1 ± 0.5	1.2 ± 0.6	1.0 ± 0.4	0.8 ± 0.4	1.3 ± 0.5
dxC:dGTP	2.6 ± 0.9	0.8 ± 0.4	0.4 ± 0.2	0.8 ± 0.4	1.3 ± 0.4	1.1 ± 0.4	1.3 ± 0.5	0.9 ± 0.4	1.1 ± 0.4
dyC:dGTP	2.5 ± 0.8	0.8 ± 0.3	0.4 ± 0.1	0.8 ± 0.4	1.1 ± 0.4	1.2 ± 0.5	1.2 ± 0.5	0.7 ± 0.3	1.2 ± 0.4

^a^ Backbone position was monitored according to P for a DNA component or Cα for a protein component.

## Supplementary Materials

Supplementary materials can be accessed at: http://www.mdpi.com/1420-3049/21/7/822/s1.

## Abbreviations

The following abbreviations are used in this manuscript:

Ala	alanine
Arg	arginine
Asn	asparagine
dA	deoxyadenosine monophosphate
dC	deoxycytidine monophosphate
DNA	deoxyribose nucleic acid
dNTP	deoxyribose nucleoside triphosphate
Dpo4	DNA polymerase IV
dG	deoxyguanosine monophosphate
dN	DNA nucleotide
dT	deoxythymidine monophosphate
dxA	expanded deoxyadenosine monophosphate
dxC	expanded deoxycytidine monophosphate
dxG	expanded deoxyguanosine monophosphate
dxT	expanded deoxythymidine monophosphate
dyA	widened deoxyadenosine monophosphate
dyC	widened deoxycytidine monophosphate
dyG	widened deoxyguanosine monophosphate
dyT	widened deoxythymidine monophosphate
εdG	1,N^2^-ethenoguanine
Glu	glutamine
Gly	glycine
Kf(*exo*–)	Klenow Fragment(*exo*–)
Lys	lysine
M_1_dG	malondialdehyde-guanine
MD	molecular dynamics
Met	methionine
Mg	magnesium
MM/GBSA	Molecular Mechanics / Generalized Born Surface Area
PdG	1,N^2^-propanoguanine
Phe	phenylalanine
RMSD	root-mean-square deviation
Ser	serine
Thr	threonine
Tyr	tyrosine
Val	valine
xDNA	expanded deoxyribose nucleic acid
yDNA	widened deoxyribose nucleic acid
