# Supplementary material for: Computational Evaluation of Nucleotide Insertion Opposite Expanded and Widened DNA by the Translesion Synthesis Polymerase Dpo4
Source: Molecules. 2016 Jun 23;21(7):822. doi: 10.3390/molecules21070822 (PMC6273265; doi:10.3390/molecules21070822)
Supplement: Supplementary file 1 [file molecules-21-00822-s001.pdf]

Supplementary Materials: Computational Evaluation of Nucleotide Insertion Opposite Expanded and Widened DNA by the Translesion Synthesis Polymerase Dpo4

Laura Albrecht, Katie A. Wilson and Stacey D. Wetmore

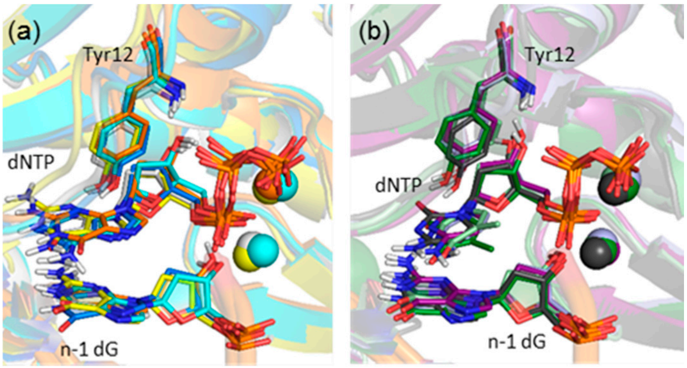

Figure S1. Overlay of representative MD structures for the Dpo4 ternary complex for the replication of (**a**) modified pyrimidines and dT (white) or (**b**) modified purines and dG (black), highlighting the interaction with Tyr12.

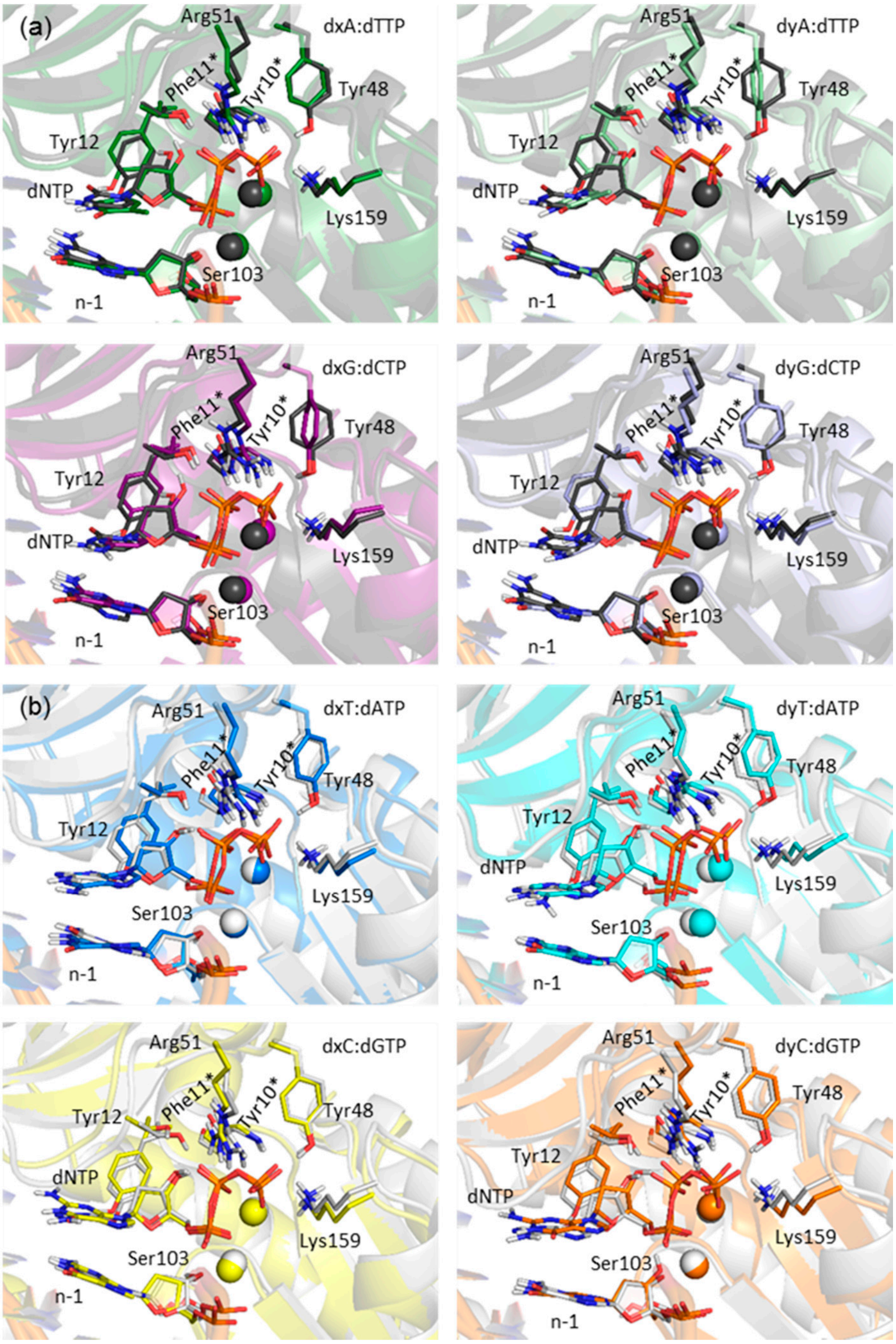

Figure S2. . Overlay of representative MD structures for the Dpo4 ternary complex for the replication of (**a**) modified purines and dG (black) or (**b**) modified pyrimidines and dT (white), highlighting the interactions with the dNTP. Only the interacting portion (side or main chain) of the amino acid residue is shown, with an * indicating that only the backbone is shown for that residue.

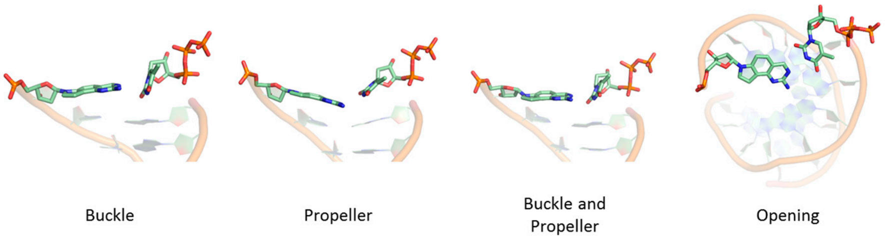

Figure S3. Snapshots taken from the simulation trajectory to show examples of base-pair distortions for the DNA helix containing dyA:dTTP in the Dpo4 ternary complex.

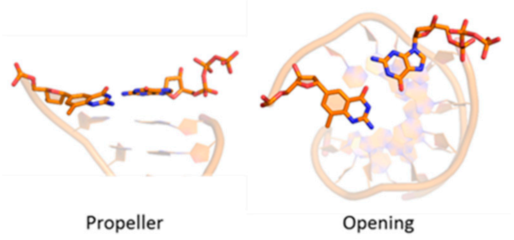

Figure S4. Snapshots taken from the simulation trajectory to show examples of base-pair distortions for the DNA helix containing dyC:dGTP in the Dpo4 ternary complex.

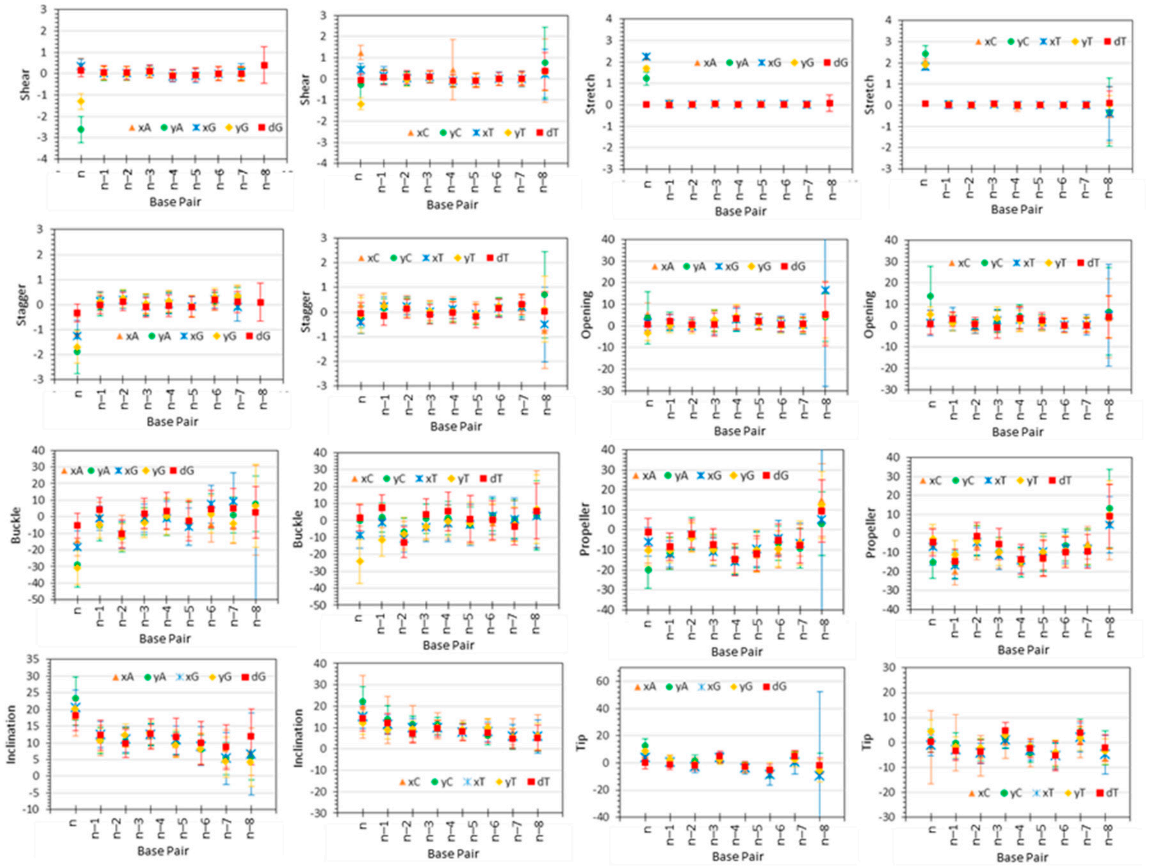

Figure S5. Average and standard deviations of base-pair parameters for the DNA, xDNA or yDNA helix in the Dpo4 ternary complex. See Figure S11 for numbering of the base pairs.

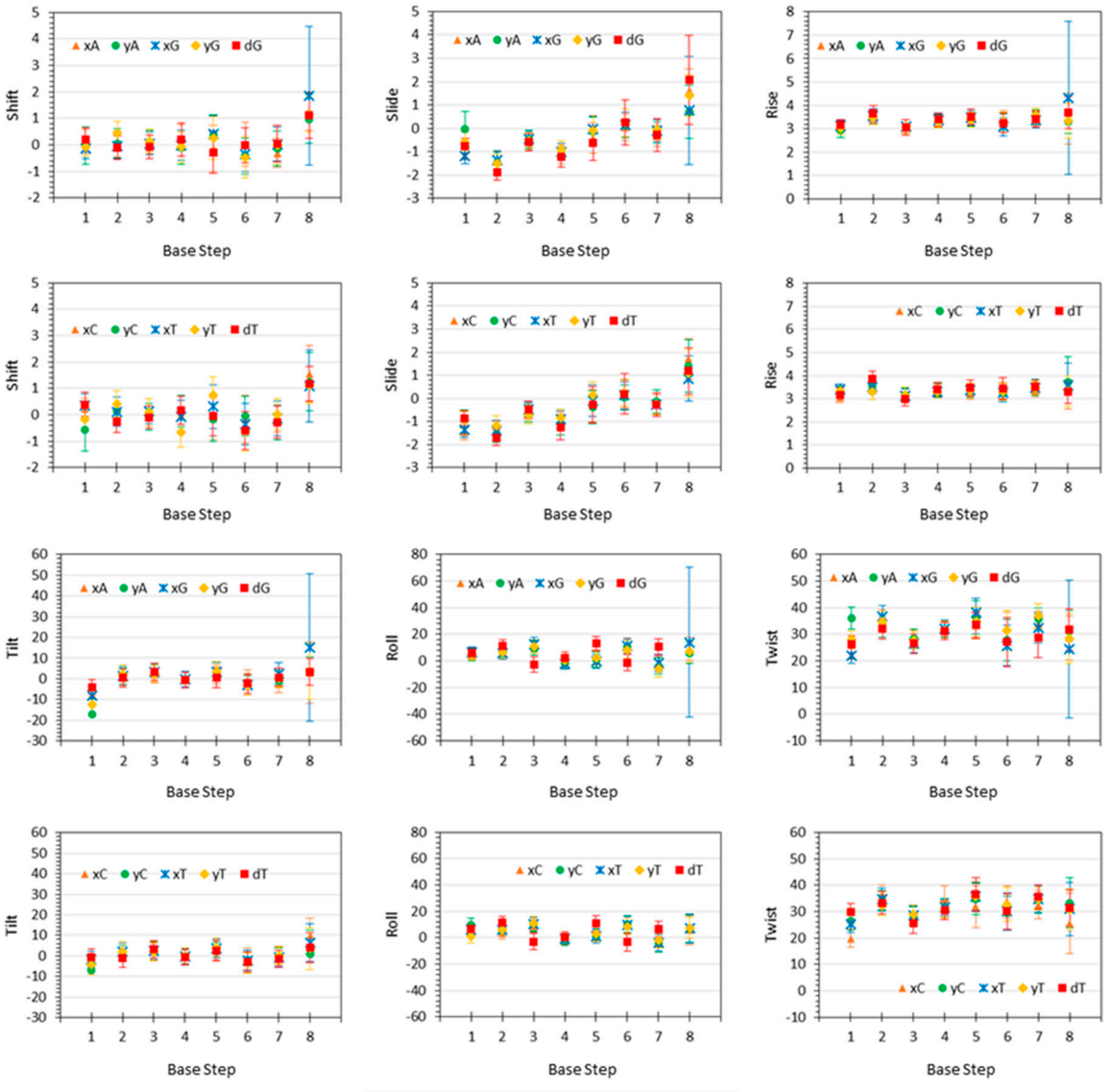

Figure S6. Average and standard deviations of base-step parameters for the DNA, xDNA or yDNA helix in the Dpo4 ternary complex. In the base-step numbering, the first step includes the dN:dNTP and n − 1 base pairs, the second step includes the n − 1 and n − 2 base pairs, and so on. See Figure S11 for numbering of the base pairs.

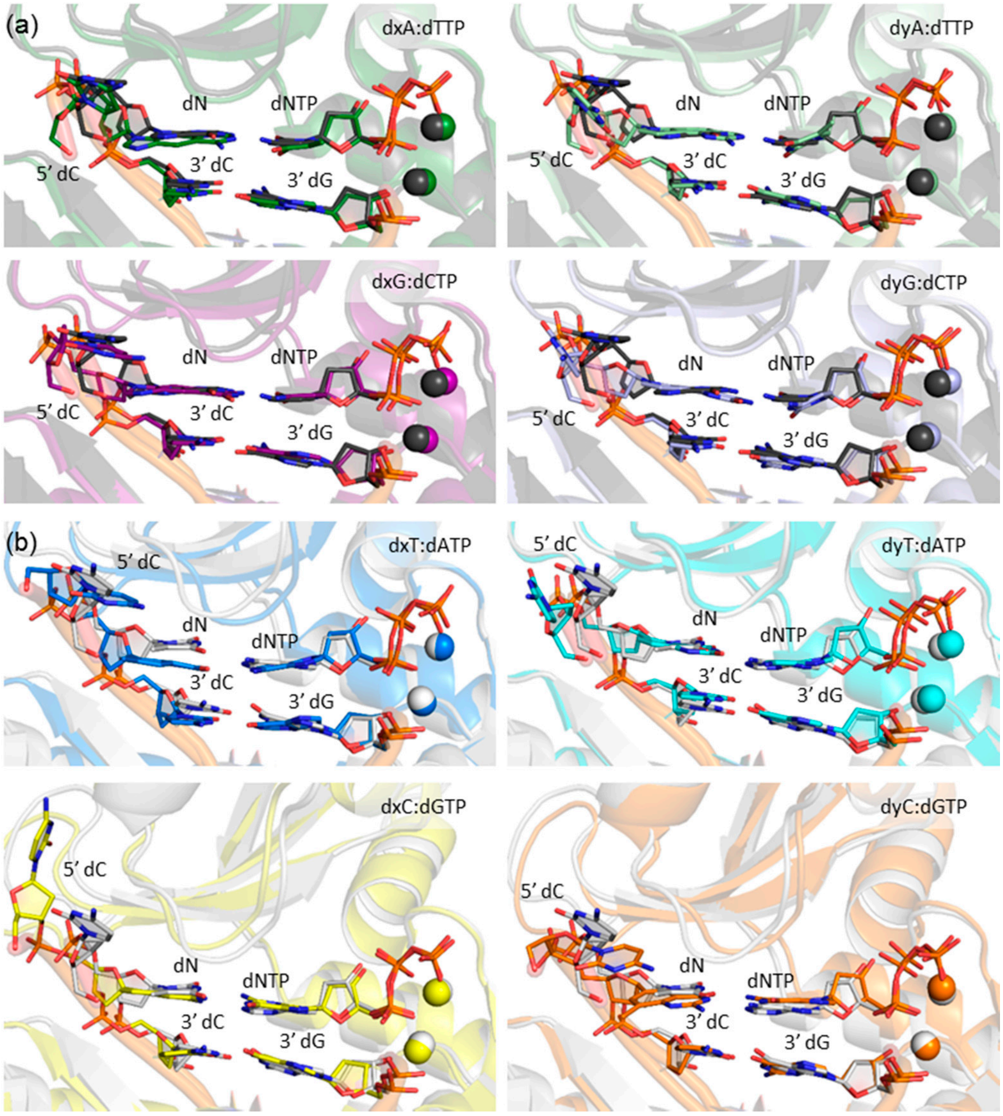

Figure S7. Overlay of representative MD structures for the Dpo4 ternary complex for the replication of (**a**) modified purines and dG (black) or (**b**) modified pyrimidines and dT (white), highlighting the structure of the dN:dNTP and n − 1 base pairs.

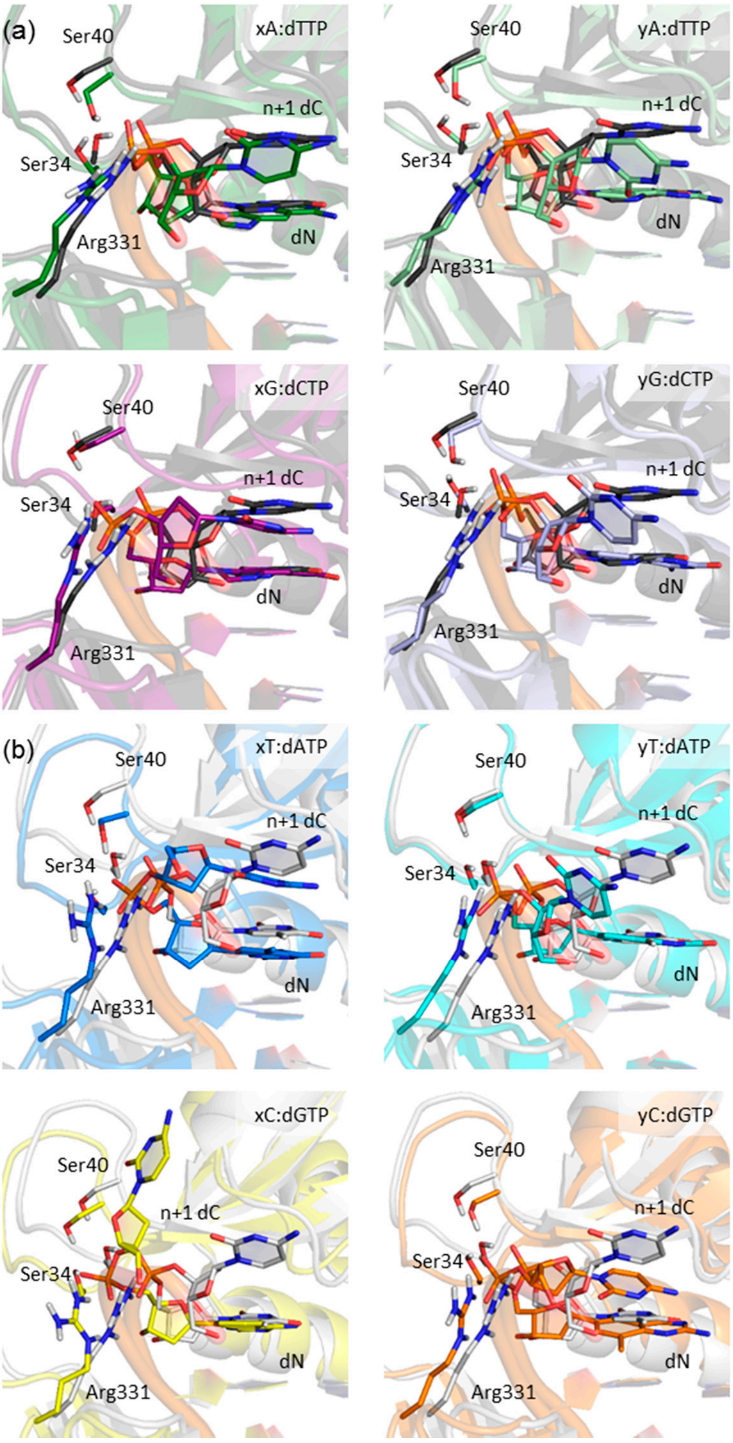

Figure S8. Overlay of representative MD structures for the Dpo4 ternary complex for the replication of (**a**) modified purines and dG (black) or (**b**) modified pyrimidines and dT (white), highlighting the interactions with the d(x,y)N backbone.

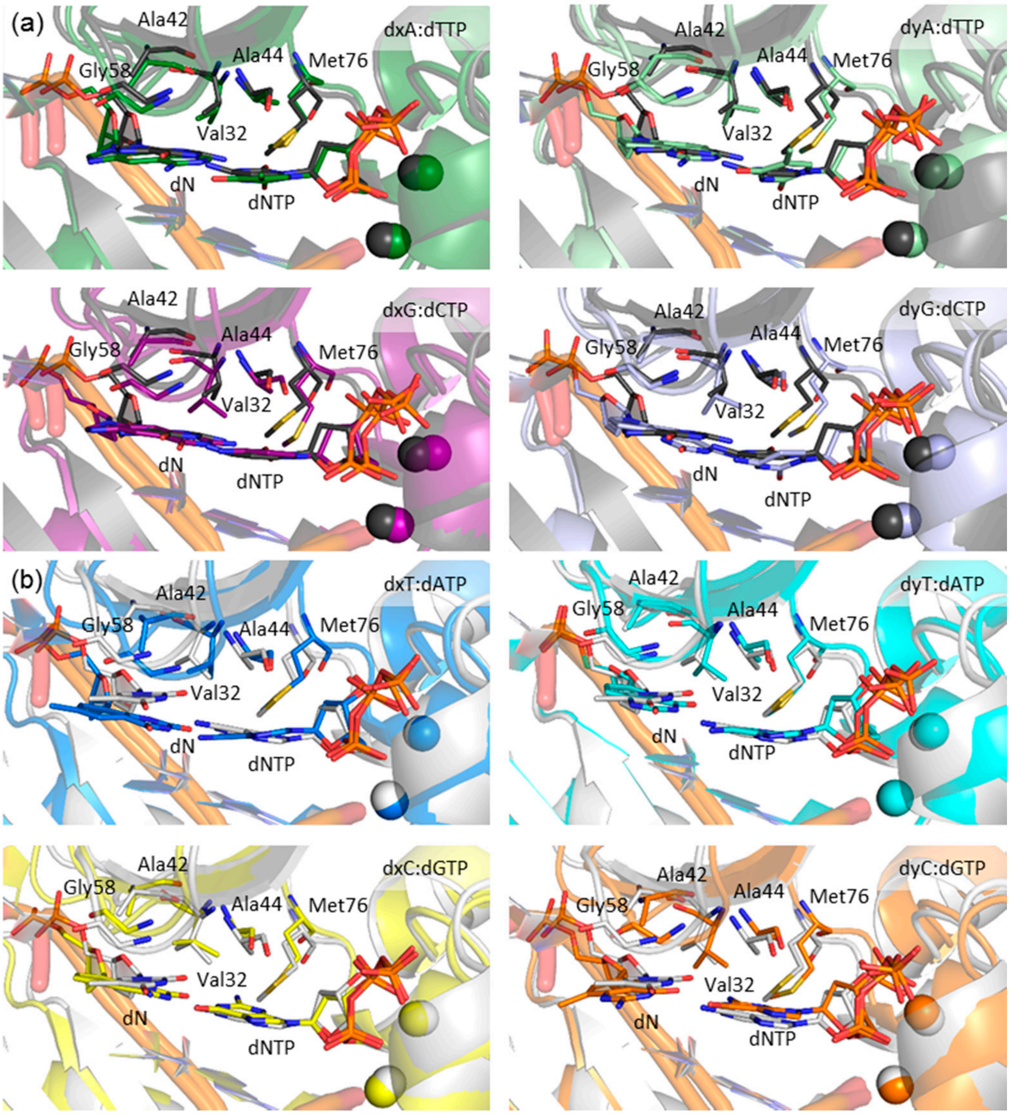

Figure S9. Overlay of representative MD structures for the Dpo4 ternary complex for the replication of (**a**) modified purines and dG (black) or (**b**) modified pyrimidines and dT (white), highlighting the structure of the dN:dNTP base pair and ceiling region.

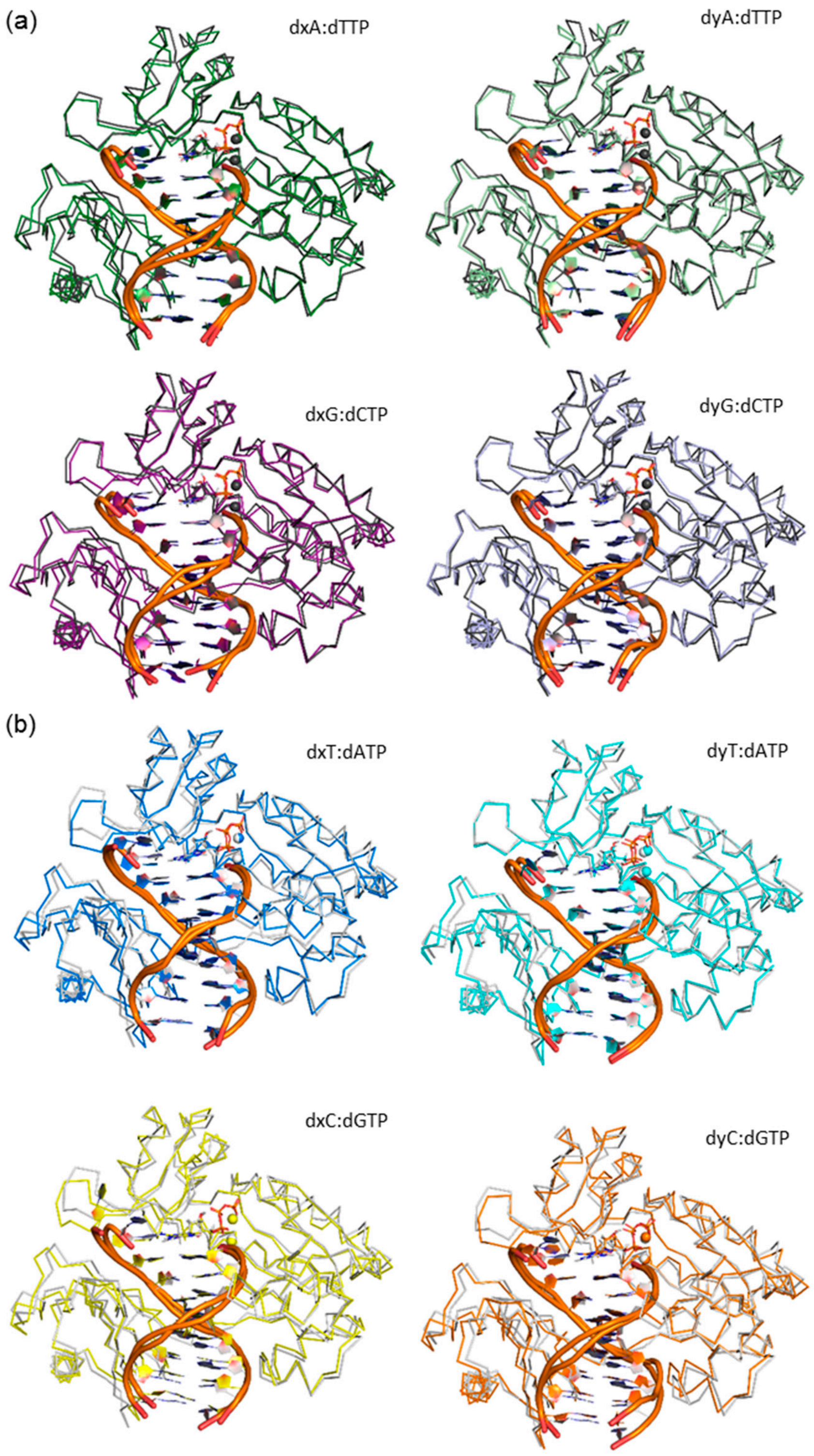

Figure S10. Overlay of representative MD structures for the Dpo4 ternary complex for the replication of (**a**) modified purines and dG (black) or (**b**) modified pyrimidines and dT (white), highlighting the overall structure of Dpo4. See Figure 2 for labeling of the domains.

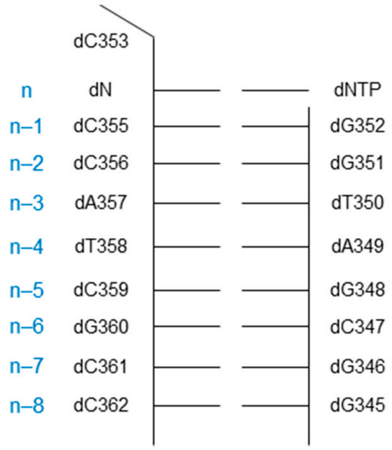

Figure S11. Numbering of the bases and base pairs in the DNA helix used throughout the analysis.

Table S1. Average MM/GBSA binding energy (kcal mol^{-1}) between individual residues and the dNTP or dN over 40 ns MD simulations of the Dpo4 ternary complex for the replication of a DNA, xDNA or yDNA base. [a,b]

Active Site Base Pair	dNTP···Thr45	dNTP···Arg51	dNTP···Tyr10	dNTP···Lys159	dNTP···Mg343	dNTP···Mg342	dN···Ser34	dN···Ser40
dG:dCTP	−11.1 ± 1.3	−43.6 ± 3.4	−14.8 ± 1.3	−56.3 ± 4.3	−227.9 ± 8.8	−90.0 ± 6.0	−4.9 ± 2.9	−1.7 ± 1.4
dT:dATP	−11.2 ± 2.0	−39.9 ± 3.6	−14.6 ± 1.3	−54.6 ± 4.7	−240.9 ± 8.2	−93.8 ± 5.7	−5.0 ± 2.7	−2.0 ± 1.9
dxA:dTTP	−10.5 ± 1.8	−41.9 ± 3.8	−14.5 ± 1.4	−56.0 ± 4.9	−225.7 ± 7.6	−85.7 ± 6.4	−4.2 ± 2.2	−5.2 ± 2.6
dyA:dTTP	−11.1 ± 1.4	−42.7 ± 4.4	−14.3 ± 1.3	−54.9 ± 5.2	−224.7 ± 7.8	−86.2 ± 6.4	−5.0 ± 2.0	−7.2 ± 2.8
dxG:dCTP	−10.2 ± 2.2	−44.2 ± 4.2	−14.7 ± 1.5	−55.9 ± 4.8	−230.2 ± 7.4	−89.9 ± 6.5	−7.4 ± 2.1	−4.1 ± 1.8
dyG:dCTP	−10.3 ± 2.3	−43.5 ± 4.3	−14.5 ± 1.4	−55.8 ± 4.7	−229.2 ± 8.0	−89.7 ± 6.5	−4.4 ± 1.3	−8.4 ± 1.6
dxT:dATP	−11.1 ± 2.3	−41 ± 4.4	−14.4 ± 1.4	−53.1 ± 5.4	−240.6 ± 7.9	−93.2 ± 5.4	−7.5 ± 2.7	−7.8 ± 1.8
dyT:dATP	−11.0 ± 2.3	−39.9 ± 4.3	−14.6 ± 1.4	−54.6 ± 4.9	−241.3 ± 7.7	−93.1 ± 5.9	−6.1 ± 2.5	−5.9 ± 2.6
dxC:dGTP	−2.9 ± 1.4	−29.2 ± 3.9	−11.2 ± 1.8	−51.0 ± 4.0	−239.4 ± 9.5	−80.3 ± 6.0	−8.3 ± 1.1	−3.9 ± 1.6
dyC:dGTP	−3.0 ± 1.3	−29.5 ± 3.4	−11.2 ± 1.8	−51.5 ± 4.3	−241.5 ± 8.3	−81.5 ± 5.6	−8.6 ± 2.4	−9.2 ± 1.4

[a] All interactions listed have a contribution of >5 kcal mol^{-1} in one or more of the simulations. [b] Interaction energy is for the DNA (including dNTP) ligand binding to the Dpo4 receptor.

Table S2. Percent occupancy, average distance and average angle for hydrogen-bonding interactions that stabilize the dNTP during 40 ns MD simulations of the Dpo4 ternary complex for the replication of a DNA, xDNA or yDNA base. [a]

Active Site Base Pair	Parameter	dNTP(O3')··· Tyr12(NH)	dNTP(Oβ)··· Phe11(NH)	dNTP(Oβγ)··· Arg51(Nη1H)	dNTP(Oγ)··· Arg51(Nη2H)	dNTP(Oγ)··· Lys159(NζH)	dNTP(Oγ)··· Tyr10(NH)	dNTP(Oγ)··· Tyr48(OH)	dNTP(Oβ)··· Thr45(OγH)
dG:dCTP	%	94	88	100	97	90	98	97	99
	Å	3.1	3.2	2.9	2.8	2.9	3.0	2.6	2.7
	°	163.0	157.5	161.4	163.0	163.5	166.2	167.7	163.7
dT:dATP	%	61	94	98	99	88	99	100	96
	Å	3.2	3.2	2.9	2.8	3.0	3.0	2.6	2.8
	°	165.4	158.7	157.7	163.5	161.9	166.9	167.4	163.4
dxA:dTTP	%	97	91	98	100	93	96	99	98
	Å	3.0	3.2	2.8	2.9	2.9	3.0	2.6	2.7
	°	163.5	157.5	163.6	161.0	162.7	166.0	168.3	162.2
dyA:dTTP	%	95	92	93	100	76	98	100	100
	Å	3.1	3.2	2.9	2.9	3.0	3.0	2.6	2.7
	°	162.7	157.2	162.0	161.8	160.5	166.4	167.7	164.6
dxG:dCTP	%	99	88	100	90	68	98	96	97
	Å	3.0	3.2	2.9	2.9	3.0	3.0	2.6	2.8
	°	162.6	157.7	161.0	160.5	160.8	166.1	167.6	161.8
dyG:dCTP	%	98	88	99	90	71	98	100	95
	Å	3.0	3.2	2.9	2.9	3.0	3.0	2.6	2.7
	°	163.3	157.2	160.3	160.7	161.4	166.2	167.6	162.3
dxC:dGTP	%	78	100		100	99	44	100	
	Å	3.2	3.0	NA [b]	2.8	2.9	3.2	2.6	NA [b]
	°	163.0	157.2		164.7	159.8	162.2	168.4	
dyC:dGTP	%	74	100		100	99	41	100	
	Å	3.2	3.0	NA [b]	2.8	2.9	3.2	2.6	NA [b]
	°	163.6	156.5		165.1	159.8	162.5	168.4	
dxT:dATP	%	62	92	99	97	61	99	100	96
	Å	3.2	3.2	2.9	2.9	3.1	2.9	2.6	2.7
	°	165.0	158.4	158.2	162.0	159.2	167.0	167.5	163.9
dyT:dATP	%	79	96	99	98	78	99	100	96
	Å	3.2	3.1	2.9	2.9	3.0	2.9	2.6	2.8
	°	165.4	158.6	158.1	162.9	159.8	166.8	167.3	163.3

[a] Hydrogen-bonding occupancies are based on a distance cutoff of <3.4 Å and an angle cutoff of >120°. [b] Not observed for >5% occupancy, with an average angle >149° and a distance of <3.4 Å.

Table S3. Percent occupancy, average distance and average angle of the hydrogen-bonding interactions between the primer strand, and the palm or thumb domain during 40 ns MD simulations of the Dpo4 ternary complex for the replication of a DNA, xDNA or yDNA base. [a]

Active Site Base Pair	Parameter	dA349(OP1)···Lys193(NζH)	dT350(OP1)···Thr190(NH)	dT350(OP1)···Thr190(OγH)	dT350(OP2)···Ile189(NH)	dT350(OP1)···Lys221(NζH)	dG352(OP1)···Lys152(NζH)	Ser103(Oγ)···dG352(O3'H)
dG:dCTP	%	36	98	100	44	39	90	91
	Å	2.8	2.9	2.6	3.1	2.9	2.8	2.8
	°	158.3	154.1	165.4	162.9	159.1	151.1	160.3
dT:dATP	%	11	75	98	58	18	95	92
	Å	2.8	2.9	2.6	3.1	2.9	2.8	2.9
	°	157.7	153.9	165.5	162.1	155.0	153.4	158.7
dxA:dTTP	%	31	99	100	51		85	80
	Å	2.8	2.9	2.6	3.1	NA [b]	2.8	2.9
	°	158.5	154.3	165.3	161.9		151.2	159.1
dyA:dTTP	%		88	99	63		83	72
	Å	NA [b]	2.9	2.6	3.1	NA [b]	2.8	2.9
	°		154.1	165.6	163.0		150.8	159.2
dxG:dCTP	%		93	100	69		86	94
	Å	NA [b]	2.9	2.6	3.1	NA [b]	2.8	2.9
	°		153.6	165.9	162.0		152.6	160.0
dyG:dCTP	%		61	98	51	6	34	97
	Å	NA [b]	2.9	2.6	3.1	2.8	2.8	2.9
	°		153.3	164.9	161.2	155.9	150.1	159.3
dxC:dGTP	%	22	95	100	53		92	85
	Å	2.8	2.9	2.6	3.1	NA [b]	2.8	2.9
	°	158.0	154.2	164.8	160.9		152.5	158.7
dyC:dGTP	%	13	99	100	59	35	92	81
	Å	2.8	2.9	2.6	3.1	2.8	2.8	2.9
	°	159.6	154.4	166.2	163.2	157.0	152.2	158.7
dxT:dATP	%	5	92	100	63		91	93
	Å	2.8	2.9	2.6	3.1	NA [b]	2.8	2.9
	°	158.8	153.9	165.6	162.5		152.9	158.8
dyT:dATP	%		67	100	83	23	98	92
	Å	NA [b]	3.0	2.6	3.0	2.9	2.8	2.9
	°		150.6	165.3	155.8	155.4	156.9	157.1

[a] Hydrogen-bonding occupancies are based on a distance cutoff of <3.4 Å and an angle cutoff of >120°. [b] Not observed for >5% occupancy, with an average angle >149° and a distance of <3.4 Å.

Table S4. Coordination of the catalytic and binding Mg^{2+} ions during 40 ns MD simulations of the Dpo4 ternary complex for the replication of a DNA, xDNA or yDNA base.

Active Site Base Pair	Catalytic Mg^{2+} ion [a]						Binding Mg^{2+} ion [a]					
	Primer(O3′)	Glu106(Oε) [b]	Asp7(Oδ1)	dNTP(Oα2)	Asp105(Oδ1)	Wat(O)	Asp7(Oδ2)	Phe8(O)	Asp105(Oδ2)	dNTP(Oα2)	dNTP(Oβ2)	dNTP(Oγ1)
dG:dCTP	100%	100%	100%	100%	100%	100%	100%	100%	100%	100%	100%	100%
dT:dTTP	100%	24.4%, 75.9%	100%	100%	100%	100%	100%	100%	100%	100%	100%	100%
dxA:dTTP	100%	29.7%, 70.1%	100%	100%	100%	100%	100%	100%	100%	99%	100%	100%
dyA:dTTP	100%	100%	100%	100%	100%	100%	100%	100%	100%	99%	100%	100%
dxG:dCTP	100%	100%	100%	100%	100%	100%	100%	100%	100%	99%	100%	100%
dyG:dCTP	100%	100%	100%	100%	100%	100%	100%	100%	100%	100%	100%	100%
dxT:dATP	100%	100%	100%	100%	100%	100%	100%	100%	100%	100%	100%	100%
dyT:dATP	100%	100%	100%	100%	100%	100%	100%	100%	100%	100%	100%	100%
dxC:dGTP	100%	100%	100%	100%	100%	100%	100%	100%	100%	100%	100%	100%
dyC:dGTP	100%	100%	100%	100%	100%	100%	100%	100%	100%	100%	100%	100%

[a] Percentage of the simulation that the distance between Mg^{2+} and the coordinating atom is <2.5 Å. See Figure 3b for the coordination of the Mg^{2+} ions. [b] Occupancy of the side chain oxygen atoms are listed separately if both share coordination to Mg^{2+}.

Table S5. Hydrogen-bonding occupancy and average geometry for the n−1 dC:dG base pair during 40 ns MD simulations of the Dpo4 ternary complex for the replication of a DNA, xDNA or yDNA base. [a]

Active Site Base Pair	C1′–C1′ [b]	dC(N4H)···dG(O6)		dC(N3)···dG(N1H)		dC(O2)···dG(N2H)	
		Occupancy	Average Geometry [c]	Occupancy	Average Geometry [c]	Occupancy	Average Geometry [c]
dG:dCTP	10.7 ± 0.2 Å	99%	2.9 Å (163.7°)	100%	2.9 Å (165.4°)	100%	2.8 Å (164.2°)
dT:dATP	10.6 ± 0.2 Å	98%	3.0 Å (163.5°)	100%	2.9 Å (165.3°)	100%	2.8 Å (163.6°)
dxA:dTTP	10.8 ± 0.2 Å	96%	3.0 Å (162.8°)	99%	3.0 Å (165.1°)	100%	2.9 Å (162.9°)
dyA:dTTP	10.7 ± 0.2 Å	98%	3.0 Å (163.3°)	100%	3.0 Å (164.4°)	100%	2.9 Å (158.8°)
dxG:dCTP	10.8 ± 0.2 Å	97%	3.0 Å (163.4°)	100%	3.0 Å (165.8°)	100%	2.9 Å (163.6°)
dyG:dCTP	10.8 ± 0.2 Å	98%	2.9 Å (162.3°)	100%	3.0 Å (165.5°)	100%	2.9 Å (161.8°)
dxT:dATP	10.7 ± 0.2 Å	98%	3.0 Å (161.9°)	98%	3.0 Å (165.2°)	100%	2.9 Å (161.9°)
dyT:dATP	10.7 ± 0.2 Å	97%	3.0 Å (161.1°)	100%	3.0 Å (163.3°)	100%	2.9 Å (159.8°)
dxC:dGTP	10.7 ± 0.2 Å	92%	3.0 Å (161.3°)	99%	3.0 Å (164.4°)	100%	2.8 Å (163.8°)
dyC:dGTP	10.7 ± 0.2 Å	96%	3.0 Å (163.3°)	100%	3.0 Å (165.2°)	100%	2.9 Å (163.1°)

[a] Hydrogen-bonding occupancies are based on a distance cutoff of <3.4 Å and an angle cutoff of >120°. [b] Average and standard deviation of the distance between dC(C1′) and dG(C1′) in the n-1 base pair. [c] Average distance and angle (in parentheses) for each hydrogen-bonding interaction.

Table S6. Average MM/GBSA binding energy (kcal mol^{-1}) between individual residues and the DNA over 40 ns MD simulations of the Dpo4 ternary complex for the replication of a DNA, xDNA or yDNA base. [a–c]

Active Site Base Pair	dG346···Arg298	dC347···Ser297	dC356···Arg247	dC356···Lys 78	dC356···Lys275	dA357···Lys275	dA357···Arg242	dT358···Arg242	dT358···Arg240	dC359···Arg240
dG:dCTP	−8.8 ± 2.3	−2.3 ± 2.5	−11.6 ± 2.7	−3.4 ± 3.5	−14.7 ± 9.4	−6.4 ± 3.8	−8.0 ± 3.6	−9.9 ± 4.9	−0.6 ± 0.9	−0.6 ± 1.2
dT:dATP	−9.5 ± 1.9	−4.5 ± 3.3	−11.6 ± 3.3	−7.2 ± 4.6	−17 ± 7.6	−8.1 ± 2.7	−9.2 ± 3	−10.4 ± 3.2	−1.1 ± 1.4	−2.0 ± 3.1
dxA:dTTP	−10.0 ± 2.3	−5.3 ± 2.7	−11.5 ± 2.9	−1.8 ± 0.9	−11.4 ± 6	−7.0 ± 4.0	−11.1 ± 3.6	−6.5 ± 2.4	−5.0 ± 3.3	−5.8 ± 4.8
dyA:dTTP	−9.8 ± 2.1	−4.9 ± 2.9	−11.1 ± 2.9	−1.7 ± 0.9	−10.1 ± 5.9	−5.3 ± 4.1	−11.4 ± 3.4	−6.1 ± 3.1	−3.7 ± 3.9	−5.7 ± 4.3
dxG:dCTP	−10.0 ± 1.7	−5.0 ± 2.8	−10.4 ± 2.7	−1.9 ± 1.4	−12 ± 6.5	−6.4 ± 3.7	−10.2 ± 3.3	−8.2 ± 4.6	−3.0 ± 3.2	−3.7 ± 4.2
dyG:dCTP	−12.7 ± 2.8	−5.4 ± 2.4	−12.6 ± 2.8	−1.3 ± 0.7	−12.4 ± 5.1	−5.6 ± 3.4	−11.6 ± 2.4	−8.4 ± 4.5	−2.7 ± 4.8	−1.4 ± 3.1
dxT:dATP	−11.4 ± 2.4	−5.8 ± 2.9	−11.3 ± 2.6	−1.9 ± 0.9	−10.0 ± 6.3	−4.7 ± 3.7	−10.0 ± 5.5	−5.3 ± 1.1	−3.5 ± 3.3	−3.6 ± 4.3
dyT:dATP	−9.7 ± 2.2	−5.8 ± 2.6	−13.6 ± 2.9	−1.7 ± 0.9	−2.4 ± 3.9	−1.3 ± 2.2	−8.9 ± 4.6	−7.0 ± 4.2	−0.5 ± 0.3	−0.6 ± 1
dxC:dGTP	−9.6 ± 1.9	−5.7 ± 2.5	−10.8 ± 2	−4.2 ± 3.7	−9.8 ± 6.3	−3.0 ± 2.3	−13.1 ± 2.5	−5.7 ± 0.7	−7.3 ± 3.1	−7.2 ± 3.5
dyC:dGTP	−9.5 ± 2.0	−4.8 ± 2.9	−12.1 ± 2.6	−2.6 ± 2.0	−7.3 ± 4.8	−5.2 ± 3.9	−7.3 ± 4.1	−5.5 ± 1.5	−0.6 ± 0.6	−0.5 ± 1.2

[a] Only residues having an interaction energy >5 kcal mol^{-1} in at least one of the simulations have been included. See Figure S11 for the numbering of the DNA helix.
[b] Interaction energy is for the DNA (including dNTP) ligand binding to the Dpo4 receptor. [c] See Table S1 for contribution of the ternary complex to dN and dNTP binding.

Table S7. Percent occupancy, average distance and average angle of the hydrogen-bonding interactions between the template strand and the finger, palm or thumb domain during 40 ns MD simulations of the Dpo4 ternary complex for the replication of a DNA, xDNA or yDNA base. [a]

Active Site Base Pair	Parameter	Gly58(O)···dC353(N4H)	Glu63(Oε)···dC353(N4H)	dN(OP1)···Ser34(OγH)	dN(OP1)···Ser40(OγH)	dC356(OP1)···Lys78(NζH)	dG360(OP1)···Lys221(NζH)	dC361(OP1)···Ala220(NH)
	%			46		13	30	28
dG:dCTP	Å	NA [b]	NA [b]	2.8	NA [b]	2.8	2.8	3.1
	°			162.4		158.3	157.1	162.0
	%			46		53		56
dT:dATP	Å	NA [b]	NA [b]	2.7	NA [b]	2.8	NA [b]	3.1
	°			164.3		155.6		162.8
	%			20	56		21	54
dxA:dTTP	Å	NA [b]	NA [b]	2.8	2.7	NA [b]	2.8	3.1
	°			163.0	161.7		157.6	160.7
	%			20	78			50
dyA:dTTP	Å	NA [b]	NA [b]	2.7	2.6	NA [b]	NA [b]	3.1
	°			164.8	161.0			160.0
	%	49		77	13		25	48
dxG:dCTP	Å	2.9	NA [b]	2.8	2.7	NA [b]	2.8	3.1
	°	160.4		162.7	161.3		157.6	160.6
	%				96			53
dyG:dCTP	Å	NA [b]	NA [b]	NA [b]	2.6	NA [b]	NA [b]	3.1
	°				162.2			162.7
	%		59	99				65
dxC:dGTP	Å	NA [b]	2.9	2.7	NA [b]	NA [b]	NA [b]	3.1
	°		155.1	162.7				163.4
	%	82		81	99		35	43
dyC:dGTP	Å	2.9	NA [b]	2.7	2.6	NA [b]	2.8	3.1
	°	160.4		165.6	165.6		161.1	159.2
	%	49		68	97			49
dxT:dATP	Å	2.9	NA [b]	2.7	2.6	NA [b]	NA [b]	3.1
	°	160.0		165.6	163.5			162.5
	%	14		53	45			67
dyT:dATP	Å	2.9	NA [b]	2.7	2.7	NA [b]	NA [b]	3.1
	°	160.7		164.2	161.5			162.9

[a] Hydrogen-bonding occupancies are based on a distance cutoff of <3.4 Å, an angle cutoff of >120° and a MM/GBSA energy cutoff of 5 kcal mol^{-1}. See Figure S11 for numbering of the DNA helix. [b] Not observed with >5% occupancy, with an average angle >149° and a distance of <3.4 Å.

Table S8. Percent occupancy, average distance and average angle of the hydrogen-bonding interactions occurring between the template strand (dN or the n+1 dC) and the little finger domain during 40 ns MD simulations of the Dpo4 ternary complex for the replication of a DNA, xDNA or yDNA base. [a]

Active Site Base Pair	Parameter	dN(O1)···Arg331(Nη2H)	dN(OP2)···Arg331(Nη2H)	dN(OP1)···Arg331(Nη2H)	dC355(OP2)···Arg332(NεH)	dC355(OP1)···Thr250(NH)	dC355(OP2)···Thr250(OγH)	dC355(OP1)···Arg247(NηH)
	%		72	30	100	48	100	12
dG:dCTP	Å	NA [b]	2.8	2.9	2.8	3.1	2.7	3.1
	°		161.2	156.7	150.7	158.3	163.9	150.9
	%		67	34	100	33	100	
dT:dATP	Å	NA [b]	2.8	2.9	2.8	3.2	2.7	NA [b]
	°		159.6	157.4	150.1	157.8	163.0	
	%		99		100	17	100	36
dxA:dTTP	Å	NA [b]	2.8	NA [b]	2.8	3.2	2.7	2.9
	°		160.2		152.0	151.1	162.4	152.6
	%	99			100	26	100	
dyA:dTTP	Å	2.8	NA [b]	NA [b]	2.8	3.2	2.8	NA [b]
	°	161.8			150.9	149.9	161.2	
	%		100		100	26	100	
dxG:dCTP	Å	NA [b]	2.8	NA [b]	2.8	3.2	2.7	NA [b]
	°		158.1		150.2	149.6	158.8	
	%		99		100	27	100	
dyG:dCTP	Å	NA [b]	2.8	NA [b]	2.8	3.2	2.8	NA [b]
	°		161.6		150.2	150.5	161.2	
	%		99		99	100	7	58
dxC:dGTP	Å	NA [b]	2.8	NA [b]	2.9	2.7	3.2	3.0
	°		159.6		150.0	161.4	151.2	155.3
	%		99		100	31	100	
dyC:dGTP	Å	NA [b]	2.8	NA [b]	2.8	3.1	2.7	NA [b]
	°		161.5		153.0	155.6	163.1	
	%		97		99	19	100	37
dxT:dATP	Å	NA [b]	2.8	NA [b]	2.9	3.2	2.7	2.9
	°		162.2		149.7	154.1	162.9	157.4
	%		99		100	17	100	
dyT:dATP	Å	NA [b]	2.8	NA [b]	2.8	3.2	2.7	NA [b]
	°		160.4		151.2	154.6	161.2	

[a] Hydrogen-bonding occupancies are based on a distance cutoff of <3.4 Å, an angle cutoff of >120° and a MM/GBSA energy cutoff of 5 kcal mol^{-1}. See Figure S11 for numbering of the DNA helix. [b] Not observed with >5% occupancy, with an average angle >149° and a distance of <3.4 Å.

Table S9. Backbone RMSD for the domain over 40 ns MD simulations of the Dpo4 ternary complex for the replication of a DNA, xDNA or yDNA base. [a,b]

Active Site Base Pair	Enzyme	Palm Domain	Finger Domain	Thumb Domain	Little Finger Domain	Tether	Finger Loop
dG:dCTP	1.0 ± 0.2	0.8 ± 0.2	1.1 ± 0.3	1.1 ± 0.2	1.1 ± 0.3	1.1 ± 0.3	0.6 ± 0.2
dT:dATP	1.1 ± 1.1	0.8 ± 0.8	1.1 ± 1.1	1.1 ± 1.1	1.1 ± 1.1	1.1 ± 1.1	0.7 ± 0.7
dxA:dTTP	0.9 ± 0.1	0.8 ± 0.1	1.0 ± 0.2	0.9 ± 0.2	1.0 ± 0.2	1.0 ± 0.2	0.7 ± 0.2
dyA:dTTP	1.0 ± 0.1	0.8 ± 0.1	1.2 ± 0.2	1.0 ± 0.2	1.2 ± 0.2	0.9 ± 0.2	0.6 ± 0.2
dxG:dCTP	1.1 ± 0.2	0.9 ± 0.2	1.2 ± 0.3	1.1 ± 0.2	1.2 ± 0.3	1.2 ± 0.2	0.7 ± 0.2
dyG:dCTP	1.0 ± 0.2	0.8 ± 0.1	1.2 ± 0.3	1.0 ± 0.2	1.1 ± 0.3	1.1 ± 0.3	0.7 ± 0.2
dxT:dATP	1.1 ± 0.2	0.9 ± 0.1	1.0 ± 0.2	1.1 ± 0.3	1.2 ± 0.3	1.3 ± 0.3	0.6 ± 0.2
dyT:dATP	1.0 ± 0.1	0.8 ± 0.1	1.1 ± 0.2	1.0 ± 0.2	1.0 ± 0.3	1.0 ± 0.2	0.7 ± 0.2
dxC:dGTP	1.1 ± 0.2	0.9 ± 0.2	1.1 ± 0.2	1.1 ± 0.3	1.1 ± 0.3	1.2 ± 0.3	0.7 ± 0.2
dyC:dGTP	1.0 ± 0.1	0.8 ± 0.1	1.0 ± 0.2	0.9 ± 0.2	1.1 ± 0.3	1.2 ± 0.3	0.7 ± 0.2

[a] Simulation Cα RMSD relative to the representative structure for the simulation. Initial alignment is based on the entire enzyme. [b] Domain residues are: palm domain (residues 1–10 and 78–166), finger domain (residues 11–77), thumb domain (residues 167–233), little finger domain (residues 244–341), tether (residues 234–343), and finger loop (residues 41–46).

Table S10. Backbone RMSD for each domain over 40 ns MD simulations of the Dpo4 ternary complex for the replication of an xDNA or yDNA base relative to the replication of natural DNA. [a,b]

Active Site Base Pair Comparison	Enzyme	Palm Domain	Finger Domain	Thumb Domain	Little Finger Domain	Tether	Finger Loop
dxA:dTTP *vs.* dG:dCTP	1.2 ± 0.1	0.9 ± 0.1	1.1 ± 0.1	1.2 ± 0.2	1.4 ± 0.3	1.1 ± 0.3	0.7 ± 0.2
dyA:dTTP *vs.* dG:dCTP	1.1 ± 0.2	0.9 ± 0.1	1.1 ± 0.2	1.2 ± 0.3	1.3 ± 0.4	1.1 ± 0.3	0.8 ± 0.2
dxG:dCTP *vs.* dG:dCTP	1.2 ± 0.2	0.9 ± 0.2	1.2 ± 0.2	1.2 ± 0.2	1.4 ± 0.3	1.1 ± 0.2	0.9 ± 0.2
dyG:dCTP *vs.* dG:dCTP	1.2 ± 0.2	0.9 ± 0.1	1.1 ± 0.2	1.4 ± 0.3	1.4 ± 0.2	1.1 ± 0.3	0.9 ± 0.2
dxT:dATP *vs.* dT:dATP	1.3 ± 0.1	1.0 ± 0.2	1.4 ± 0.3	1.1 ± 0.2	1.5 ± 0.3	1.4 ± 0.3	0.7 ± 0.2
dyT:dATP *vs.* dT:dATP	1.2 ± 0.1	0.9 ± 0.2	1.2 ± 0.2	1.1 ± 0.2	1.4 ± 0.2	1.2 ± 0.2	0.8 ± 0.2
dxC:dGTP *vs.* dT:dATP	1.3 ± 0.2	0.9 ± 0.2	1.3 ± 0.2	1.1 ± 0.2	1.7 ± 0.3	1.6 ± 0.4	0.8 ± 0.2
dyC:dGTP *vs.* dT:dATP	1.3 ± 0.2	0.9 ± 0.2	1.3 ± 0.2	1.1 ± 0.2	1.6 ± 0.3	1.3 ± 0.3	0.8 ± 0.1

[a] Simulation Cα RMSD relative to the representative structure from the corresponding natural DNA simulation. Initial alignment is based on the entire enzyme. [b] Domain residues are: palm domain (residues 1–10 and 78–166), finger domain (residues 11–77), thumb domain (residues 167–233), little finger domain (residues 244–341), tether (residues 234–343), and finger loop (residues 41–46).

Table S11. Percent occupancy, average distance and average angle of the hydrogen-bonding interactions between the template strand (not dN or the n+1 dC) and the little finger domain during 40 ns MD simulations of the Dpo4 ternary complex for the replication of a DNA, xDNA or yDNA base. [a]

Active Site Base Pair	Parameter	dC356(OP1)··· Lys275(NζH)	dC356(OP2)··· Ile248(NH)	dC356(OP1)··· Arg247(Nη1H)	dC356(OP1)··· Arg247(NεH)	dC356(O3')··· Lys275(NζH)	dA357(OP2)··· Gly246(NH)	dA357(OP1)··· Gly246(NH)	dA357(OP2)··· Arg336(Nη1H)	dA357(O5')··· Arg336(Nη2H)	dT358(OP2)··· Ser244(OγH)
	%	52	98	33		45		43	100	92	69
dG:dCTP	Å	2.8	3.0	2.9	NA [b]	3.0	NA [b]	3.0	2.8	3.0	2.7
	°	155.6	159.0	158.5		151.9162		160.1	158.6	152.4	163.3
	%	58	94			54		55	100	94	87
dT:dATP	Å	2.8	3.0	NA [b]	NA [b]	3.1	NA [b]	3.1	3.8	3.0	2.7
	°	154.0	159.8			150.2		159.9	160.9	154.1	163.5
	%	26	98	38			80	17	100	86	53
dxA:dTTP	Å	2.8	2.9	2.8	NA [b]	NA [b]	3.0	3.0	2.8	3.0	2.7
	°	150.2	157.6	159.4			149.8	160.8	156.5	151.2	163.8
	%	46	99					11	99	93	85
dyA:dTTP	Å	2.8	2.9	NA [b]	NA [b]	NA [b]	NA [b]	3.1	2.8	3.0	2.7
	°	152.0	159.6					158.0	157.4	149.3	164.3
	%	40	97			39		36	100	93	88
dxG:dCTP	Å	2.8	3.0	NA [b]	NA [b]	3.1	NA [b]	3.0	2.8	3.0	2.7
	°	149.7	158.9			149.2		159.2	159.8	151.2	164.8
	%	66	99	25	21			7	100		83
dyG:dCTP	Å	2.8	2.9	2.9	2.8	NA [b]	NA [b]	3.1	2.8	NA [b]	2.7
	°	153.9	158.9	158.8	152.2			157.7	157.4		164.6
	%	58	99						100		82
dxC:dGTP	Å	2.8	2.9	NA [b]	NA [b]	NA [b]	NA [b]	NA [b]	2.8	NA [b]	2.7
	°	150.1	160.0						155.2		164.5
	%	5	99					40	100	86	72
dyC:dGTP	Å	2.8	3.0	NA [b]	NA [b]	NA [b]	NA [b]	3.0	2.8	3.0	2.7
	°	150.2	162.9					160.9	157.0	150.3	164.1
	%	49	99					7	100		74
dxT:dATP	Å	2.8	2.9	NA [b]	NA [b]	NA [b]	NA [b]	3.0	2.8	NA [b]	2.7
	°	152.5	161.4					160.5	155.9		163.9
	%	5	100						100		96
dyT:dATP	Å	2.8	2.9	NA [b]	NA [b]	NA [b]	NA [b]	NA [b]	2.8	NA [b]	2.7
	°	154.6	163.5						156.4		165.2

[a] Hydrogen-bonding occupancies are based on a distance cutoff of <3.4 Å, an angle cutoff of >120° and a MM/GBSA energy cutoff of 5 kcal mol^{-1}. See Figure S11 for the numbering of DNA helix. [b] Not observed with >5% occupancy, an average angle >149° and a hydrogen-bond distance of <3.4 Å.

Table S12. Percent occupancy, average distance and average angle of the hydrogen-bonding interactions occurring between the primer strand and the little finger domain during 40 ns MD simulations of the Dpo4 ternary complex for the replication of a DNA, xDNA or yDNA base. [a]

Active Site Base Pair	Parameter	dC346(OP2)··· Gly299(NH)	dC346(OP1)··· Arg298(Nη1H)	dC347(OP2)···Ser297(NH)	dC347(OP1)··· Arg298(Nη1H)	dC347(OP2)··· Ser297(OγH)
dG:dCTP	%	79		51	20	18
	Å	2.9	NA [b]	3.0	3.0	2.8
	°	156.7		153.3	150.9	163.1
dT:dATP	%	84			37	56
	Å	3.0	NA [b]	NA [b]	3.0	2.7
	°	154.9			150.3	164.1
dxA:dTTP	%	87	12		14	68
	Å	2.9	3.0	NA [b]	3.0	2.7
	°	156.0	151.9		149.6	163.5
dyA:dTTP	%	96			37	58
	Å	3.0	NA [b]	NA [b]	3.0	2.8
	°	155.7			150.4	164.2
dxG:dCTP	%	93			26	56
	Å	3.0	NA [b]	NA [b]	3.0	2.748
	°	154.5			149.3	164.8
dyG:dCTP	%	94	65			71
	Å	2.9	2.8	NA [b]	NA [b]	2.8
	°	154.8	156.9			164.0
dxC:dGTP	%	84				70
	Å	2.9	NA [b]	NA [b]	NA [b]	2.7
	°	153.8				163.8
dyC:dGTP	%	81			32	58
	Å	3.0	NA [b]	NA [b]	3.0	2.8
	°	150.7			149.3	164.4
dxT:dATP	%	97	36			68
	Å	3.0	2.8	NA [b]	NA [b]	2.7
	°	156.7	156.6			164.4
dyT:dATP	%	92				75
	Å	3.0	NA [b]	NA [b]	NA [b]	2.7
	°	152.2				164.9

[a] Hydrogen-bonding occupancies are based on a distance cutoff of <3.4 Å, an angle cutoff of >120° and a MM/GBSA energy cutoff of 5 kcal mol^{-1}. See Figure S11 for the numbering of DNA helix. [b] Not observed with >5% occupancy, with an average angle <149° and a hydrogen–bond distance of <3.4 Å.

Table S13. Percent occupancy, average distance and average angle of the hydrogen-bonding interactions between the template strand and the tether domain during 40 ns MD simulations of the Dpo4 ternary complex for the replication of a DNA, xDNA or yDNA base. [a]

Active Site Base Pair	Parameter	dA357(OP1)···Arg242(Nη1H)	dT358(OP1)···Lys243(NH)	dT358(OP1)···Arg242(Nη1H)	dT358(OP1)···Arg242(NεH)	dC359(OP1)···Arg240(NηH)	dC359(OP2)···Arg240(NηH)	dC359(OP2)···Lys243(NζH)	dG360(OP1)···Lys221(NζH)
	%		79					68	
dG:dCTP	Å	NA [b]	2.9	NA [b]	NA [b]	NA [b]	NA [b]	2.8	NA [b]
	°		160.3					155.7	
	%		92	77		19		71	
dT:dATP	Å	NA [b]	2.9	2.9	NA [b]	2.9	NA [b]	2.8	NA [b]
	°		158.3	153.6		150.9		156.5	
	%	59	62			88	35	72	
dxA:dTTP	Å	2.8	2.9	NA [b]	NA [b]	2.9	3.0	2.8	NA [b]
	°	157.9	162.0			151.9	155.6	157.5	
	%	76	90			48	22	66	
dyA:dTTP	Å	2.8	2.9	NA [b]	NA [b]	2.9	3.0	2.8	NA [b]
	°	157.0	161.6			153.5	152.0	156.7	
	%	48	92			32	16	68	
dxG:dCTP	Å	2.8	2.9	NA [b]	NA [b]	2.9	3.0	2.8	NA [b]
	°	157.9	161.7			153.8	156.2	157.0	
	%	62	88			7	5	69	
dyG:dCTP	Å	2.8	2.9	NA [b]	NA [b]	3.0	2.9	2.8	NA [b]
	°	156.9	161.5			151.2	151.7	156.5	
	%	98	87			73	21	77	
dxC:dGTP	Å	2.8	2.9	NA [b]	NA [b]	2.9	3.0	2.8	NA [b]
	°	154.9	160.8			155.7	156.4	158.3	
	%	33	79					65	35
dyC:dGTP	Å	2.8	2.9	NA [b]	NA [b]	NA [b]	NA [b]	2.8	2.8
	°	156.1	161.2					155.9	157.0
	%	69	81			31	14	72	
dxT:dATP	Å	2.8	2.9	NA [b]	NA [b]	2.9	2.9	2.8	NA [b]
	°	158.1	161.5			156.8	155.9	157.1	
	%	42	97		22			79	
dyT:dATP	Å	2.8	2.9	NA [b]	2.9	NA [b]	NA [b]	2.8	NA [b]
	°	154.3	159.3		149.9			156.6	

[a] Hydrogen-bonding occupancies are based on a distance cutoff of <3.4 Å, an angle cutoff of >120° and a MM/GBSA energy cutoff of 5 kcal mol^{-1}. See Figure S11 for the numbering of DNA helix. [b] Not observed with >5% occupancy, with an average angle >149° and a distance of <3.4 Å.

Table S14. Relative pairwise MM/GBSA energies (kcal mol^{-1}) between individual residues in the finger or palm domains and DNA in the Dpo4 ternary complex for the replication of an xDNA or yDNA base compared to a DNA base. [a]

	Finger [c]					Palm [d]				Mg^{2+} ions		
**Active Site Base Pair [b]**	**dNTP··· Thr45**	**dNTP··· Arg51**	**dN··· Ser34**	**dN··· Ser40**	**Total[e]**	**dNTP··· Tyr10**	**dNTP··· Lys159**	**dC356··· Lys78**	**Total [e]**	**dNTP··· Binding Mg^{2+}**	**dNTP··· Catalytic Mg^{2+}**	**Total [e]**
dxA:dTTP	0.5	1.7	0.7	−3.5	5.0	0.3	0.3	1.6	1.7	2.2	4.2	7.9
dyA:dTTP	−0.1	0.9	−0.1	−5.4	0.4	0.4	1.4	1.7	3.4	3.2	3.8	8.1
dxG:dCTP	0.8	−0.6	−2.4	−2.4	0.4	0.1	0.4	1.5	1.8	−2.3	0.1	−3.2
dyG:dCTP	0.8	0.1	0.6	−6.7	−0.3	0.2	0.5	2.1	3.0	−1.3	0.3	0.5
dxT:dATP	0.1	−1.1	−2.4	−5.8	−4.8	0.2	1.4	5.3	7.2	0.4	0.6	2.4
dyT:dATP	0.3	0.0	−1.1	−3.8	−0.2	0.0	0.0	5.5	5.4	−0.4	0.7	2.6
dxC:dGTP	8.3	10.7	−3.3	−1.9	16.8	3.3	3.6	3.0	10.4	1.6	13.5	13.0
dyC:dGTP	8.2	10.4	−3.6	−7.1	10.8	3.4	3.1	4.6	11.2	−0.6	12.3	6.0

[a] Interaction energies represent the DNA (including dNTP) ligand binding to the Dpo4 receptor. See Figure S11 for numbering of DNA helix. [b] Energies for the (x,y)-purines are relative to the dG:dCTP simulation, while energies for the (x,y)-pyrimidines are relative to the dT:dATP simulation. [c] Finger domain includes residues 11-77. [d] Palm domain includes residues 1-10 and 78-166. [e] Sum of the energy changes listed for each domain.

Table S15. Relative pairwise MM/GBSA energies (kcal mol^{-1}) between individual residues in the little finger domain or tether and DNA in the Dpo4 ternary replication complex for the replication of an xDNA or yDNA base compared to a DNA base. [a]

	Little Finger [c]						Tether [d]				
**Active Site Base Pair [b]**	**dC347···Ser297**	**dG346···Arg298**	**dC356··· Arg247**	**dA357···Lys275**	**dC356··· Lys275**	**Total [d]**	**dC359···Arg240**	**dT358···Arg240**	**dA357···Arg242**	**dT358··· Arg242**	**Total [d]**
dxA:dTTP	−3.0	−1.2	0.1	−0.6	3.3	6.9	−5.3	−4.4	−3.1	3.4	−9.5
dyA:dTTP	−2.6	−1.0	0.5	1.1	4.6	7.5	−5.1	−3.1	−3.4	3.8	−6.9
dxG:dCTP	−2.6	−1.2	1.2	−0.1	2.7	3.6	−3.1	−2.4	−2.2	1.8	−5.2
dyG:dCTP	−3.1	−3.9	−1.0	0.8	2.3	6.9	−0.8	−2.1	−3.6	1.5	−5.7
dxT:dATP	−1.3	−1.9	0.3	3.4	7.0	12.7	−1.7	−2.3	−0.8	5.1	0.3
dyT:dATP	−1.2	−0.2	−2.0	6.8	14.6	25.4	1.3	0.6	0.3	3.5	5.5
dxC:dGTP	−1.1	0.0	0.8	5.1	7.2	18.2	−5.2	−6.1	−3.9	4.8	−11.8
dyC:dGTP	−0.3	0.0	−0.5	2.8	9.7	19.0	1.4	0.5	2.0	5.0	8.8

[a] Interaction energies represent the DNA (including dNTP) ligand binding to the Dpo4 receptor. See Figure S11 for numbering of DNA helix. [b] Energies for the (x,y)-purines are relative to the dG:dCTP simulation, while energies for the (x,y)-pyrimidines are relative to the dT:dATP simulation. [c] Little finger domain includes residues 244-341. [d] Tether domain includes residues 234-243. [e] Sum of the energy changes listed for each domain.

Table S16. Partial charges and AMBER atom types for dxA.

Atom	Atom Type	Charge
P	P	1.2203
O1P	O2	−0.7930
O2P	O2	−0.7930
O5'	OS	−0.5023
O3'	OS	−0.5517
C9	CB	0.0173
N13	N*	−0.0282
C8	CB	0.3745
C10	CA	−0.2996
C12	CK	0.2018
N11	NB	−0.5996
C7	CM	−0.3694
C4	CA	0.4045
H10	HA	0.2203
H12	H5	0.1504
C5	CA	−0.1689
H7	HA	0.2148
N3	NC	−0.7680
C6	CA	0.7721
C2	CQ	0.6517
N1	NC	−0.7718
N6	N2	−0.9135
H2	H5	0.0299
H61	H	0.3904
H62	H	0.3904
C1'	CT	0.0357
O4'	OS	−0.3774
H1'	H2	0.0871
C2'	CT	0.0334
C4'	CT	0.0703
C3'	CT	0.2297
H2'	HC	0.0321
H2''	HC	0.0321
C5'	CI	0.0048
H4'	H1	0.0826
H3'	H1	0.0988
H5'	H1	0.0957
H5''	H1	0.0957

Table S17. Partial charges and AMBER (GAFF) atom types for dxC.

Atom	Atom Type	Charge
P	P	1.2203
O1P	O2	−0.7933
O2P	O2	−0.7933
O5'	OS	−0.5055
O3'	OS	−0.5410
C9	CA	−0.1337
C8	CA	−0.1852
C10	CA	0.0248
H9	HA	0.1465
C7	CA	−0.2243
H8	HA	0.1553
C6	CM	0.1356
C5	CM	−0.0272
H7	HA	0.1690
N1	(n)	−0.4042
C4	CA	0.6350
C2	C	0.8084
H1	H	0.2934
N3	NC	−0.6918
N4	N2	−0.8888
O2	O	−0.6342
H41	H	0.3944
H42	H	0.3944
C1'	CT	0.0349
O4'	OS	−0.4029
H1'	H1	0.0736
C2'	CT	0.0323
C4'	CT	0.0902
C3'	CT	0.1572
H2'	HC	0.0285
H2''	HC	0.0285
C5'	CI	0.0217
H4'	H1	0.0879
H3'	H1	0.1191
H5'	H1	0.0872
H5''	H1	0.0872

Table S18. Partial charges and AMBER atom types for dxG.

Atom	Atom Type	Charge
P	P	1.2202
O1P	O2	−0.7930
O2P	O2	−0.7930
O5'	OS	−0.5015
O3'	OS	−0.5505
C9	CB	0.0307
N13	N*	−0.0002
C8	CB	0.3273
C10	CM	−0.3157

C12	CK	0.1462
N11	NB	−0.5672
C7	CM	−0.3101
C4	CA	0.2659
H10	HA	0.2133
H12	H5	0.1582
C5	CM	−0.0765
H7	HA	0.2264
N3	NC	−0.6638
C6	C	0.6017
C2	CA	0.7654
N1	NA	−0.5240
O6	O	−0.5869
N2	N2	−0.8938
H1	H	0.3445
H21	H	0.3792
H22	H	0.3792
C1'	CT	0.0528
O4'	OS	−0.3756
H1'	H2	0.0760
C2'	CT	0.0358
C4'	CT	0.0577
C3'	CT	0.2220
H2'	HC	0.0312
C5'	CI	0.0043
H4'	H1	0.0886
H3'	H1	0.1014
H5'	H1	0.0963
H5''	H1	0.0963
H2''	HC	0.0312

Table S19. Partial charges and AMBER (GAFF) atom types for dxT.

Atom	Atom Type	Charge
P	P	1.2198
O1P	O2	−0.7939
O2P	O2	−0.7939
O5'	OS	−0.4937
O3'	OS	−0.5341
C9	CA	−0.0892
C8	CA	0.0329
C10	CM	−0.0643
H9	HA	0.1261
C7	CA	−0.2007
C11	CT	−0.1140
C6	CM	−0.0227
C5	CM	0.0013
H7	HA	0.1894
H111	HC	0.0492
H112	HC	0.0492
H113	HC	0.0492
N1	(n)	−0.1257
C4	C	0.4750
C2	C	0.4112
H1	H	0.2568
N3	n	−0.2887
O4	O	−0.5492
O2	O	−0.5500
H3	H	0.3001
C1'	CT	0.0417
O4'	OS	−0.3755
H1'	H1	0.0910
C2'	CT	−0.0249
C4'	CT	0.0399
C3'	CT	0.1516
H2'1	HC	0.0516
H2'2	HC	0.0516
C5'	CI	−0.0296
H4'	H1	0.1127
H7	H1	0.1356
H5'1	H1	0.1071
H5'2	H1	0.1071

Table S20. Partial charges and AMBER atom types for dyA.

Atom	Atom Type	Charge
P	P	1.2200
O1P	O2	−0.7931
O2P	O2	−0.7931
O5'	OS	−0.4957
O3'	OS	−0.5530
C8	CB	0.0741
N13	N*	−0.0035
C7	CB	−0.0453
C9	CA	−0.1167
C12	CA	−0.1882
C4	CA	0.4082
C11	C*	−0.2746
C10	CA	−0.3976
H9	HA	0.1556
H12	H4	0.2003
C3	CA	−0.0583
N5	NC	−0.7103
H11	HA	0.1822

H10	HA	0.1992
C2	CA	0.2768
C6	CQ	0.9537
N1	NC	−0.6997
H2	H4	0.1016
N6	N2	−0.9391
H61	H	0.3865
H62	H	0.3865
C1'	CT	0.0786
O4'	OS	−0.3863
H1'	H2	0.0795
C2'	CT	0.0509
C4'	CT	0.0718
C3'	CT	0.2110
H2'	HC	0.0235
H2''	HC	0.0235
C5'	CI	0.0037
H4'	H1	0.0830
H7	H1	0.0979
H5'	H1	0.0932
H5''	H1	0.0932

Table S21. Partial charges and AMBER atom types for dyC.

Atom	Atom Type	Charge
P	P	1.2199
O1P	O2	−0.7936
O2P	O2	−0.7936
O5'	OS	−0.4945
O3'	OS	−0.5428
C8	CA	−0.1803
C9	CA	0.0191
C7	CA	−0.0045
H8	HA	0.1446
C10	CA	−0.1951
C6	CM	0.1450
C11	CT	−0.0847
C1	CM	−0.1320
H10	HA	0.1844
N5	NA	−0.5301
H111	HC	0.0376
H112	HC	0.0376
H113	HC	0.0376
C2	C	0.8211
C4	CA	0.8036
H5	H	0.3608
N3	NC	−0.7468
O2	O	−0.6235
N4	N2	−0.9013
H41	H	0.3835
H41	H	0.3835
C1'	CT	0.0896
O4'	OS	−0.3939
H1'	H1	0.0742
C2'	CT	−0.0751
C4'	CT	0.0643
C3'	CT	0.1659
H2'	HC	0.0592
H2''	HC	0.0592
C5'	CI	−0.0136
H4'	H1	0.0964
H3'	H1	0.1249
H5'	H1	0.0967
H5''	H1	0.0967

Table S22. Partial charges and AMBER (GAFF) atom types for dyG.

Atom	Atom Type	Charge
P	P	1.2201
O1P	O2	−0.7932
O2P	O2	−0.7932
O5'	OS	−0.4971
O3'	OS	−0.5494
C8	CB	0.1156
N13	N*	−0.0154
C7	CB	−0.0563
C9	CA	−0.1747
C12	CA	−0.2231
C4	CA	0.3457
C11	C*	−0.2218
C10	CA	−0.3632
H9	HA	0.1572
H12	H4	0.2113
C3	CM	−0.0056
N5	NC	−0.643
H11	HA	0.1735
H10	HA	0.1975
C2	CM	0.3678
C6	C	0.7369
N1	(n)	−0.3802
N2	N2	−0.8056
O6	O	−0.6415
H1	H	0.3217
H21	H	0.3903

H22	H	0.3903
C1'	CT	0.0851
O4'	OS	−0.3855
H1'	H2	0.0859
C2'	CT	0.0439
C4'	CT	0.0767
C3'	CT	0.1922
H2'	HC	0.0289
H2''	HC	0.0289
C5'	CI	0.0003
H4'	H1	0.0854
H7	H1	0.1044
H5'	H1	0.0946
H5''	H1	0.0946

Table S23. Partial charges and AMBER (GAFF) atom types for dyT.

Atom	Atom Type	Charge
P	P	1.2198
O1P	O2	−0.7936
O2P	O2	−0.7936
O5'	OS	−0.492
O3'	OS	−0.5426
C8	CA	−0.0142
C9	CA	−0.0072
C7	CM	−0.3239
H8	HA	0.1246
C10	CA	−0.1204
C6	CM	0.0413
H7	HA	0.1854
C1	CM	0.0658
H10	HA	0.1622
N5	(n)	−0.2268
C2	C	0.3812
C4	C	0.423
H5	H	0.2952
N3	(n)	−0.2162
O2	O	−0.524
O4	O	−0.5442
H3	H	0.2816
H1'	H1	0.0858
C1'	CT	0.0218
O4'	OS	−0.3882
C2'	CT	−0.0435
C4'	CT	0.0574
C3'	CT	0.1873
H2'	HC	0.052
H2''	HC	0.052
C5'	CI	−0.0253
H4'	H1	0.1003
H5	H1	0.117
H5'	H1	0.101
H5''	H1	0.101

Table S24. RMSD over 40 ns MD simulations of the Dpo4 ternary complex for the replication of a DNA, xDNA or yDNA base. [a]

Active Site Base Pair	RMSD
dG:dCTP	1.3 ± 0.2
dT:dATP	1.5 ± 0.3
dxA:dTTP	1.3 ± 0.2
dyA:dTTP	1.3 ± 0.2
dxG:dCTP	1.2 ± 0.2
dyG:dCTP	1.4 ± 0.2
dxT:dATP	1.2 ± 0.2
dyT:dATP	1.1 ± 0.1
dxC:dGTP	1.2 ± 0.2
dyC:dGTP	1.6 ± 0.2

[a] RMSD is relative to the first frame in the respective simulation.
